# HELLO: improved neural network architectures and methodologies for small variant calling

**DOI:** 10.1186/s12859-021-04311-4

**Published:** 2021-08-14

**Authors:** Anand Ramachandran, Steven S. Lumetta, Eric W. Klee, Deming Chen

**Affiliations:** 1 Department of Electrical and Computer Engineering, University of Illinois At Urbana-Champaign, Urbana, IL 61801 USA; 2 Biomedical Statistics and Informatics, Department of Quantitative Health Sciences, Mayo Clinic, Rochester, MN USA

**Keywords:** Variant calling, Deep learning, Deep neural networks, Illumina, PacBio, Hybrid variant calling

## Abstract

**Background:**

Modern Next Generation- and Third Generation- Sequencing methods such as Illumina and PacBio Circular Consensus Sequencing platforms provide accurate sequencing data. Parallel developments in Deep Learning have enabled the application of Deep Neural Networks to variant calling, surpassing the accuracy of classical approaches in many settings. DeepVariant, arguably the most popular among such methods, transforms the problem of variant calling into one of image recognition where a Deep Neural Network analyzes sequencing data that is formatted as images, achieving high accuracy. In this paper, we explore an alternative approach to designing Deep Neural Networks for variant calling, where we use meticulously designed Deep Neural Network architectures and customized variant inference functions that account for the underlying nature of sequencing data instead of converting the problem to one of image recognition.

**Results:**

Results from 27 whole-genome variant calling experiments spanning Illumina, PacBio and hybrid Illumina-PacBio settings suggest that our method allows vastly smaller Deep Neural Networks to outperform the Inception-v3 architecture used in DeepVariant for indel and substitution-type variant calls. For example, our method reduces the number of indel call errors by up to 18%, 55% and 65% for Illumina, PacBio and hybrid Illumina-PacBio variant calling respectively, compared to a similarly trained DeepVariant pipeline. In these cases, our models are between 7 and 14 times smaller.

**Conclusions:**

We believe that the improved accuracy and problem-specific customization of our models will enable more accurate pipelines and further method development in the field. HELLO is available at https://github.com/anands-repo/hello

**Supplementary Information:**

The online version contains supplementary material available at 10.1186/s12859-021-04311-4.

## Background

Variant calling has wide range of applicability in modern bioinformatics. Discovering the underlying genetic traits of Mendelian diseases [1], understanding the individual’s susceptibility to cancer[2, 3] and study of genetic diversity to help strategize crop-breeding methods [4] are just a few of these applications. Substitutions and small insertions and deletions (indels), which account for most mutations in a typical human genome [5], are of great interest in many of these studies.

Advances in sequencing technology have enabled probing genomes at a higher resolution than before. Sequencing reads from Next Generation Sequencing (NGS) platforms such as Illumina are typically of a few hundred bases long and are highly accurate. Recent advances in Third Generation Sequencing (TGS) have given rise to sequencing reads which are long (thousands to tens of thousands of bases) with improved accuracy, such as from the Pacific Biosciences’ Circular Consensus Sequencing (CCS) platform. The primary errors in Illumina sequencing reads are the substitution type [6], and while the average base quality is high, these sequencing reads with errors cannot be mapped accurately (or at all) [7] everywhere in a reference sequence due to their short length, which is a drawback. PacBio CCS reads are not affected by mapping and mappability issues as severely because of their longer length. While these reads also have low average error-rates, the errors are of indel type and are highly context specific [8], hence it may be hard to call certain types of indel variants even when there is sufficient read coverage. Small variant calling using data from these two sequencing technologies provide high accuracy results. Since both sequencing platforms have different error profiles, it is also beneficial to combine data from the two platforms to perform hybrid variant calling that compensates each other’s weaknesses.

Traditionally, variant calling methods have used probabilistic models of sequencing errors and sequencing read alignment to determine likelihoods of variation at genomic loci from sequencing data. For instance, the pair-Hidden Markov Model (HMM) has been used [9] to determine alignment probabilities of reads to different candidate haplotypes at a site leading to the determination of the best haplotypes at the site. DeepVariant [10], which demonstrated how Deep Learning may be applied to variant calling, on the other hand uses a Deep Neural Network (DNN) that makes predictions for candidate alternative alleles and pairs of candidate alternative alleles at a site. These predictions are then converted into variant calls using additional algorithms to filter, sort and rank the different alleles at the site. DeepVariant outperforms traditional methods in many published experimental settings for both indels and substitutions and shows that a sophisticated and general-purpose pattern recognizer such as a DNN with a large parameter space can better capture sequencing noise and alignment characteristics at genomic loci than traditional statistical models which use a small number of parameters but a larger number of domain assumptions. We also note another Deep Learning-based tool, Clairvoyant [11], a small variant caller which runs rapidly for noisy long-read technologies, which can call SNVs and indels of length up to 4 bp. Clairvoyant uses a single input summarizing all the data aligning to a location, and uses a DNN to predict variant alleles and lengths from this input. In this article, we look at high-accuracy small variant calling from both short and newer and higher accuracy long-read technologies, and the methods compared here make calls for the full range of small variant lengths (1–50 bp) by making predictions for each candidate allele based on analyzing their associated read supports.

While DeepVariant has shown the ability of DNNs to effectively call variants, it is important to challenge the fundamental approaches used in DeepVariant in order to make improvements in variant calling. Such efforts become increasingly relevant as more and more difficult-to-call sites are benchmarked. For example, recent efforts [12] added more than 300,000 new Single Nucleotide Variants (SNVs) and 50,000 new indels to previous ground-truth sets in difficult regions of the genome. Continual improvements of such benchmarks can upend assumptions regarding best performing methods since methods developed previously may not work as effectively for newly revealed segments. In addition, smaller models that can be trained with smaller training sets are helpful for applications where obtaining new training data may not be easy, such as for new target organisms, or where a customized pipeline is desired that is tailored to a specific lab’s variant calling pipeline and quality control heuristics.

Machine Learning models use *inductive biases*, or simplifying assumptions, which constrain them to learning specific types of patterns that generalize well beyond the training set. Image recognition DNNs such as those used by DeepVariant (which uses the Inception-v3 DNN) encode certain inductive biases that work well for the problem of image recognition, such as stationarity and locality [13]. However, the DNN in DeepVariant encodes scant assumptions specific to sequencing data in the model’s architecture. Not encoding stronger assumptions about the application can be helpful in the case where the data is complex, such as images of general objects or animals, where it is hard to manually and exhaustively define individual elements of the problem. However, in a problem such as variant calling, some basic elements of the data are easily and precisely defined. We know that sequencing data is in the form of read sequences, and that a variant call supported by a larger number of reads is more likely to be correct than a variant call supported by a smaller number of reads. The objectives difficult to engineer or define exhaustively in this case are models of sequencing and mapping errors, and how to factor these errors in deciding thresholds for calling a variant. The question then is whether we can constrain or encourage a Deep Learning method to expend its learning prowess on the unknown aspects of the problem, rather than spend it on learning almost everything about the problem from an abstract representation of the input data such as images. Incorporating genome sequencing-specific inductive biases into the Deep Learning machinery can achieve this goal.

The concept of *relational inductive bias* [14], where the DNN designer explicitly creates structures in the architecture representing elements of the problem as well as their interrelationship, has been studied before. The basic objects in the variant calling setting are sequencing reads and candidate alleles. Taking our cue from these, we build a DNN architecture that recognizes reads and alleles as the elements of the problem, and introduces specific constructs encoding the relationships of reads to alleles, as well as the relationship of one allele to other alleles. Thus, our method, called HELLO (**H**ybrid and stand-alone **E**stimation of sma**LL** gen**O**mic variants), built for small variant calling, accounts for the nature and structure of genome sequencing data and tailors the DNN model to the problem at hand. Explicitly representing these aspects of the problem in the DNN can reduce the learning burden of the machinery as compared to, say, an image recognizer that lacks a notion of reads, or doesn’t use the concept requiring that evidence from multiple reads should add up, and instead operates with assumptions well-suited to another domain. Through this encoding of structure of the data into a DNN, we are effectively combining elements of hand-engineering approaches with the Deep Learning approach. Finally, we introduce a principled way in which DNN predictions for each allele at a site can be combined to produce the variant call result through log likelihood maximization instead of resorting to complex algorithms or a second machine learning method. This approach allows the entire framework to also be extended to polyploid cases with no changes to the underlying DNN.

We performed 27 whole-genome variant calling experiments at various coverage points for Illumina, PacBio and hybrid variant calling using data from the Genome-In-A-Bottle (GIAB) [15, 16] repository. We compared our methods to DeepVariant and Genome Analysis ToolKit (GATK) using these datasets. Our models are up to 14 × smaller in terms of parameter count compared to DeepVariant but performed similarly to or better than DeepVariant in different settings. In the following sections, we describe in detail, our experimental results and methodology.

## Results

In this section, we describe the tools, datasets, experimental setup, and evaluation results.

### Tool versions and training hardware

In addition to HELLO, we performed experiments using DeepVariant version 1.1 [17] and GATK version 4.2.0.0 [18]. Both HELLO and DeepVariant were trained using the same datasets in all our experiments. For training the DeepVariant models, we used Google Cloud Tensor Processing Unit (TPU). For training HELLO, we used our local cluster which contains machines with IBM POWER8 and POWER9 CPUs and NVIDIA Tesla V100 and Tesla K80 Graphics Processing Units (GPUs). GATK was run for Illumina and PacBio datasets using the Docker image downloaded from Dockerhub.

### Training datasets and training overview

For training HELLO and DeepVariant, we used the GIAB HG002 whole genome BAM files, and prepared alignments at multiple coverage points to enable the DNNs to make robust predictions across coverage points. For both Illumina and PacBio sequencing reads, we emphasized lower coverage data more than higher coverage data in the training set since variant calling is more challenging at lower coverages, but also quite valuable given that both sequencing and computation costs are lower at lower coverage. This relationship is true especially for PacBio reads since they are more expensive and have valid use cases for structural variant calling even at lower coverages. With these points in mind, we downloaded 300 × coverage Illumina alignment data from the GIAB repository. Following conversion from Binary Alignment Map (BAM) format to FASTQ files, we split the FASTQ files into non-overlapping paired-end FASTQ files of coverages 15x, 20x, 25x, 30x, 40x, and 50 × through random partitioning. For PacBio, we downloaded 52 × coverage CCS sequencing data for HG002 available from GIAB, and prepared non-overlapping BAM files through random partitioning at fractions of 1/3 and 1/6 to obtain three whole genome datasets at coverages approximately 52x, 17x, and 9x.

Each set of paired-end FASTQ files prepared for Illumina was aligned to the GRCh38 human reference build using the Burrows-Wheeler Aligner (BWA) tool [19]. For HELLO, we performed indel realignment using GATK 3.8.1. DeepVariant performs indel realignment internally using its own implementation, so we directly used the alignment files without realignment.

Haplotagging is a procedure where reads estimated to be from the same haplotypes are tagged identically. This process can be performed using the Whatshap tool [20] for PacBio sequencing reads. For DeepVariant, we trained the model on haplotagged PacBio alignment files since DeepVariant is known to perform the best when haplotags are present in the data for PacBio reads. For DeepVariant, options needed specially for PacBio data were added to the training dataset generation script. For PacBio datasets, we trained two HELLO models, one which calls variants from PacBio data without haplotags, and another which calls variants from alignment files which are haplotagged. We found the haplotag model to perform better and present those results in this article relegating the other result to the Additional file 1.

For hybrid training data, for each chromosome and for each Illumina dataset described above, we randomly selected a PacBio dataset described above and prepared hybrid training datasets using the two. The same chromosome and alignment file combinations were used for HELLO and DeepVariant. In addition, for DeepVariant we used the “samtools merge” [21] command to produce a single BAM file with both PacBio and Illumina reads, and used Picard [22] to apply the same read group to all the reads (both Illumina and PacBio) as needed for DeepVariant hybrid training. HELLO simply accepts PacBio and Illumina BAM files as inputs. For DeepVariant, the generated training datasets were shuffled as required for the tool before training.

Chromosomes 21 and 22 were not used for training HELLO and DeepVariant (the two chromosomes were held out completely and were not used for gradient descent nor for determining the best model). For both HELLO and DeepVariant, we designated a checkpoint interval (or epoch) as one complete pass through the training dataset. We performed, by default, 20 epochs of training for both methods. For DeepVariant hybrid training, we found the 20^th^ epoch to have the highest validation accuracy, and we continued training for another 8 epochs when we found a pre-final epoch to give the best validation accuracy.

For labeling the training examples, we used GIAB benchmark variant set 4.2 for the GRCh38 reference. Details on data preparation are released with the Additional file 1.

### Evaluation datasets

We performed three evaluations as follows.

- GIAB HG003 whole genome data with GIAB benchmark variant set 4.2.1
- GIAB HG003 chromosomes 21, and 22 with GIAB benchmark variant set 4.2.1
- GIAB HG001 whole genome data with GIAB benchmark variant set 3.3.2 (the latest version available for this genome)

GIAB benchmark variant set 4.2.1 is the latest iteration of ground truth variant sets from the *Genome In A Bottle* consortium. It adds approximately 300,000 SNVs and 50,000 indels not included previously and reveals many regions in the reference that are difficult to call using short read sequencing technologies like Illumina. This variant calling set is only available currently for the trio HG002 (son), HG003 (father) and HG004 (mother). Using this ground-truth set for training exposes the models to challenging patterns in the reference producing more robust models. Using this ground-truth set for evaluation provides a more comprehensive picture of a tool’s capabilities. Hence it is important to test our models using this latest ground-truth version. So, we selected GIAB HG003 as one of the datasets on which to report variant calling accuracies.

HG002 and HG003 are genomes of different but related individuals. To quantify the similarity between the two genomes from a variant calling perspective, we used the hap.py tool [23], designating HG003’s truth Variant Call Format (VCF) file as the truth VCF and HG002’s truth VCF as the query VCF restricting analysis to the confident regions in HG003. We got an F1 score of only 0.532 for indels and 0.579 for SNVs. Compared to this, HELLO produces variant calls with an F1 score of 0.99 for indels and 0.999 for SNVs with 30 × coverage PacBio data for HG003. This means that a model trained on HG002 cannot make such highly accurate predictions on HG003 simply through memorization (or overfitting) of the training data. However, while it seems very likely that a highly accurate model is not making predictions on one genome by overfitting on the other, there may still be an influence of their genetic relation in the variant calling accuracy. Hence it is important to examine variant calling accuracy where this genetic relation is not a factor.

To address this concern, we separately examine variant calling accuracy on GIAB HG003 chromosomes 21 and 22. As described before, chromosomes 21 and 22 were completely held out from the training procedure. Hence the models are not aware of the variants in chromosomes 21 and 22 of HG002 (the son), and the fact that HG002 and HG003 are genetically related does not influence variant calling accuracies for chromosomes 21 and 22 for HG003 (the father). In addition, using HG003 allows us to evaluate accuracies using the latest GIAB ground-truth set, which has many important qualities, as described before.

Additionally, we also present results on GIAB HG001 whole genome data. HG001 is not closely related to the individual HG002. For HG001, we use the GIAB benchmark set 3.3.2, which is the latest available GIAB truth set for that genome. As indicated before, this ground-truth set does not contain some of the challenging sites in the latest GIAB release.

For HG001 and HG003, we downloaded 300 × alignment files from the GIAB repository for Illumina. We subsampled the BAM files using samtools with different random seeds to 20x, 30x, 40x, and 50 × coverage alignment files for HG003 and to 20 × and 30 × alignment files for HG001. We converted each alignment file into paired-end FASTQ files and aligned them to the GRCh38 reference build using BWA. For GATK, we performed Base Quality Score Recalibration (BQSR) using known Single Nucleotide Polymorphism (SNP) and indel sets provided in the GATK resource bundle, and for HELLO we performed indel realignment using GATK 3.8.1. For HELLO and DeepVariant, single entry-point scripts run the complete variant call flow. For GATK, we launched haplotype calling, variant scoring, and variant filtering in succession as listed in the best practices webpage [24].

For PacBio, we downloaded HG003 60 × coverage CCS alignments. From this dataset, we prepared 15x, 30x, and 60 × coverage alignment files through subsampling (where applicable using samtools view), BAM to FASTQ conversion, and alignment with pbmm2 [25, 26]. For HG001, we downloaded 30 × coverage CCS alignments, and followed the same steps as for HG003 for preparing alignment files at 15 × and 30 × coverages. For HELLO, and DeepVariant, we performed haplotagging with the Whatshap tool [20]. For GATK, we did not find detailed recommendations from Broad Institute regarding how to call variants using PacBio reads, but prior art has described running GATK for PacBio reads [8], so we followed these recommendations.

For hybrid variant calling, we used the samtools merge command to merge each Illumina dataset described above to each PacBio dataset described above to produce 16 input files for DeepVariant. We used Picard tools to apply the same read group to all reads. HELLO accepts two different BAM files from two technologies. GATK doesn’t support hybrid variant calling as described in this paper yet.

Additional details on data preparation and variant calling are released with the Additional file 1.

### Evaluation results

We summarize the main results of the paper below.

#### GIAB HG003 whole genome variant calling

Tables 1, 2 show the results of calling variants for Illumina sequencing reads from the three methods we tested. As may be seen at lower coverages, all tools call indels at a lower accuracy than SNVs based on Precision and Recall values. The accuracy of calling indels increases significantly with coverage with many times fewer errors being called at higher coverages.

**Table 1:** Indel results for HG003 Whole Genome Sequencing (WGS), Illumina

	GATK			DeepVariant			HELLO		
	Precision	Recall	Errors	Precision	Recall	Errors	Precision	Recall	Errors
20x	0.977132	0.962505	30,699	0.980459	0.971863	24,345	0.986638	0.973921	20,052
30x	0.987019	0.981851	15,934	0.991070	0.987324	11,067	0.994046	0.987738	9284
40x	0.990826	0.988121	10,801	0.994484	0.991528	7164	0.996235	0.990973	6516
50x	**0.992820**	**0.991235**	**8191**	**0.996153**	**0.993191**	**5451**	**0.997263**	**0.992274**	**5325**

Values in bold indicate the best operating point for each tool for the corresponding metric

**Table 2:** SNV results for HG003 WGS, Illumina

	GATK			DeepVariant			HELLO		
	Precision	Recall	Errors	Precision	Recall	Errors	Precision	Recall	Errors
20x	**0.993089**	0.987367	64,904	0.994664	0.990906	47,956	0.996837	0.990922	40,679
30x	0.992641	0.992285	50,157	0.996820	0.993576	31,928	0.998147	0.993197	28,776
40x	0.992119	0.993382	48,286	0.997644	**0.994059**	27,584	0.998533	**0.993424**	26,742
50x	0.991823	**0.993881**	**47,633**	**0.998125**	0.994017	**26,125**	**0.998761**	0.993395	**26,082**

Values in bold indicate the best operating point for each tool for the corresponding metric

**Fig. 1:**
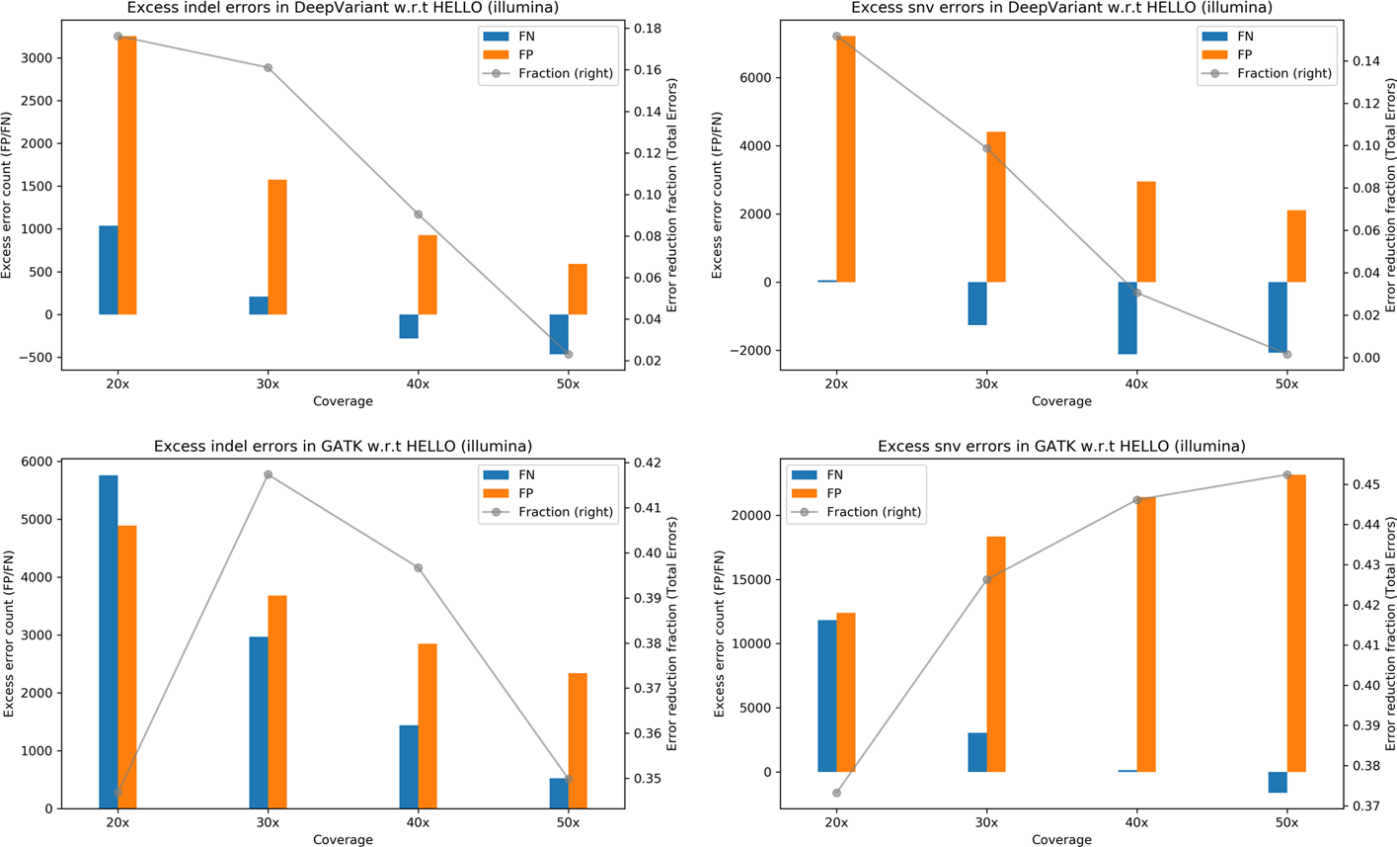
Excess errors in DeepVariant and GATK variant calls compared to HELLO for HG003 Illumina Whole Genome Sequencing (WGS) data. Positive values indicate better performance by HELLO over competitor. Bars reference the left y-axis and indicate differences in FP and FN counts between tool (GATK or DeepVariant) and HELLO. Formally, let ${\text{N}}_{{{\text{FP}}}}^{{\text{T}}} ,{\text{ N}}_{{{\text{FN}}}}^{{\text{T}}} , \in _{{\text{T}}} = \left( {{\text{N}}_{{{\text{FP}}}}^{{\text{T}}} + {\text{N}}_{{{\text{FN}}}}^{{\text{T}}} } \right)$ represent, respectively, the false positive count, false negative count, and total error count for tool ${\text{T}}$ at a given coverage point. Then, the value of the bar, marked “FP”, for that coverage point for tool ${\text{T}}$, is ${\text{N}}_{{{\text{FP}}}}^{{\text{T}}} - {\text{N}}_{{{\text{FP}}}}^{{{\text{HELLO}}}} ,$ and the value of the bar marked “FN” is ${\text{N}}_{{{\text{FN}}}}^{{\text{T}}} - {\text{N}}_{{{\text{FN}}}}^{{{\text{HELLO}}}}$. The line plots reference the right y-axis, and indicate the differences in total error count as a fraction of total errors of the worse performing method at each coverage point. Formally, for the line plots for tool ${\text{T}}$, the value of a point in the line plot is $\left( {\in_{{\text{T}}} - \in{}_{{{\text{HELLO}}}} } \right)/{\text{max}}\left( {\in_{{\text{T}}} ,{ }\in_{{{\text{HELLO}}}} } \right).$ This scheme is followed for Figs. 1, 2, 3, 4, 5, 6, 7, 8, 9

To visualize the performance of GATK and DeepVariant relative to HELLO, we prepared plots shown in Fig. 1. Each plot indicates the number of excess errors in GATK or DeepVariant with respect to HELLO. The bars indicate the absolute differences in the numbers of False Negative (FN) and False Positive (FP) calls and the lines indicate the excess total errors made by each tool as a fraction of the total number of errors called by the worse performing tool.

For indels, HELLO improves upon the number of errors in GATK by more than 30% for all cases. The improvement with respect to DeepVariant is more than 15% for 20 × and 30 × coverage data. At higher coverages, the advantage HELLO holds over DeepVariant steadily decreases. In all cases, HELLO makes fewer indel call errors than either method. Regarding SNV calls, HELLO improves upon GATK’s number of errors by more than 35% in all cases due mainly to fewer FP calls. Compared to DeepVariant, HELLO makes fewer erroneous calls in all cases, with improvements up to 15.17%.

Tables 3, 4 indicate the results for PacBio variant calling. The accuracy of calling SNVs is higher than that of calling indels in all cases. Doubling the coverage from 15 × to 30 × results in reduction of errors by approximately 2 × to 3 × for SNV calls for all methods. However, increasing coverage further doesn’t seem to have as much effect. For GATK, increasing coverage results in only modest improvements for indel calls. For DeepVariant and HELLO, however, significant reductions in indel errors are observed at 30 × with respect to 15 × and at 60 × with respect to 30 × coverage data.

**Table 3:** Indel results for HG003 WGS, PacBio

	GATK			DeepVariant			HELLO		
	Precision	Recall	Errors	Precision	Recall	Errors	Precision	Recall	Errors
15x	0.860057	0.858831	144,331	0.949591	0.947211	52,916	0.969548	0.962226	34,847
30x	0.891429	0.889284	112,742	0.980414	0.982694	19,026	0.990916	0.990159	9720
60x	**0.897505**	**0.899685**	**104,621**	**0.991986**	**0.992145**	**8172**	**0.996676**	**0.996115**	**3702**

Values in bold indicate the best operating point for each tool for the corresponding metric

**Table 4:** SNV results for HG003 WGS, PacBio

	GATK			DeepVariant			HELLO		
	Precision	Recall	Errors	Precision	Recall	Errors	Precision	Recall	Errors
15x	0.996702	0.990138	43,719	0.998137	0.994880	23,218	0.998536	0.995063	21,286
30x	0.997521	0.995412	23,501	0.999092	**0.998774**	7101	0.999470	**0.998794**	5776
60x	**0.997561**	**0.995716**	**22,357**	**0.999405**	0.998650	**6471**	**0.999636**	**0.998794**	**5223**

Values in bold indicate the best operating point for each tool for the corresponding metric

It is interesting to look at GATK’s performance given that GATK is not optimized for PacBio sequencing data. The fact that even under this situation GATK produces relatively high accuracy for SNVs compared to indels is reflective of the dominant error type in PacBio sequencing data, which are indel type errors. The relative paucity of substitution type errors in PacBio data hides the difference in error modeling to a large extent; on the other hand, the incognizance of the accurate indel error model of PacBio sequencing by GATK results in significantly lower indel call accuracy and an inability to utilize higher coverage to improve the situation significantly.

**Fig. 2:**
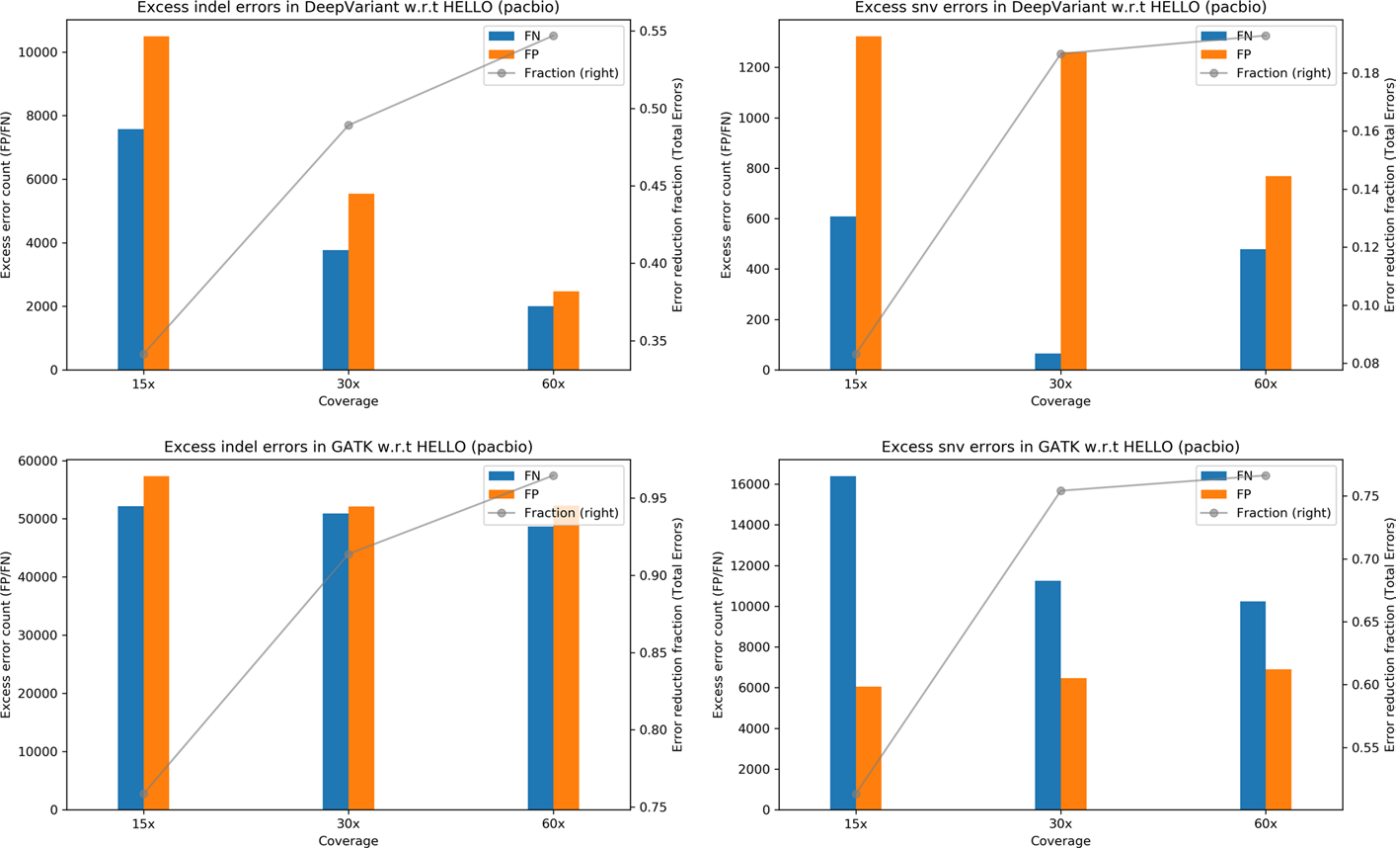
Excess errors in DeepVariant and GATK variant calls compared to HELLO for HG003 PacBio WGS data. Positive values indicate better performance by HELLO over competitor. Bars reference the left y-axis and indicate differences in FP and FN counts between tool (GATK or DeepVariant) and HELLO. The line plots reference the right y-axis, and indicate the differences in total error count as a fraction of total errors of the worse performing method at each coverage point. Please refer to Fig. 1 for additional explanation

To better visualize relative performance of the tools, we provide plots in Fig. 2 which indicate excess errors in GATK and DeepVariant compared to HELLO. HELLO makes approximately 35%—55% fewer indel errors compared to DeepVariant. Improvements are seen in both FN and FP. In the case of SNVs, HELLO makes approximately up to 20% fewer errors compared to DeepVariant. Compared to GATK, HELLO makes approximately 75–95% fewer indel errors, and approximately 50–75% fewer SNV errors. HELLO’s lead increases with coverage in both cases. For PacBio, in all cases, HELLO makes fewer errors than the compared methods.

Tables 5, 6 summarize the results of calling variants in the hybrid Illumina-PacBio case. Hybrid variant calling is interesting because coupling data from two different sequencing platforms provides an opportunity to exploit any complementarity of properties of the two types of data—for example, Illumina sequencing reads face mapping issues in certain parts of the genome and PacBio reads have context-dependent and systematic indel errors.

**Table 5:** Indel results for HG003 WGS, Hybrid

	DeepVariant			HELLO		
	Precision	Recall	Errors	Precision	Recall	Errors
20 × 15x	0.987302	0.988139	12,661	0.994011	0.993572	6381
20 × 30x	0.989490	0.991368	9894	0.996103	0.995849	4137
20 × 60x	0.989477	0.993166	9012	0.997052	0.996892	3114
30 × 15x	0.993222	0.993308	6939	0.996341	0.995970	3951
30 × 30x	0.993277	0.994482	6324	0.997269	0.997177	2856
30 × 60x	0.992232	0.995110	6570	0.997664	0.997566	2453
40 × 15x	0.995176	0.995084	5016	0.997139	0.996838	3095
40 × 30x	0.995033	0.995756	4755	0.997790	0.997649	2345
40 × 60x	0.993734	0.995974	5338	0.998024	0.997841	2125
50 × 15x	**0.996197**	0.995881	4077	0.997726	0.997257	2576
50 × 30x	0.995982	**0.996470**	**3895**	0.998047	0.997816	2126
50 × 60x	0.994690	0.996468	4582	**0.998192**	**0.998016**	**1949**

Values in bold indicate the best operating point for each tool for the corresponding metric

**Table 6:** SNV results for HG003 WGS, Hybrid

	DeepVariant			HELLO		
	Precision	Recall	Errors	Precision	Recall	Errors
20 × 15x	0.998807	0.998758	8104	0.998937	0.998999	6872
20 × 30x	0.998882	0.998790	7748	0.999233	0.999186	5266
20 × 60x	0.999035	0.998691	7565	0.999418	0.999234	4487
30 × 15x	0.999043	0.998848	7018	0.999061	0.999022	6382
30 × 30x	0.999081	0.998938	6594	0.999306	0.999185	5022
30 × 60x	0.999151	0.998820	6752	0.999443	**0.999248**	4358
40 × 15x	0.999185	0.998838	6579	0.999165	0.998970	6209
40 × 30x	0.999193	0.999006	5993	0.999354	0.999170	4912
40 × 60x	0.999223	0.998893	6269	0.999473	0.999242	4277
50 × 15x	**0.999286**	0.998796	6383	0.999243	0.998915	6131
50 × 30x	0.999272	**0.999035**	**5635**	0.999387	0.999130	4937
50 × 60x	0.999244	0.998932	6071	**0.999500**	0.999235	**4211**

Values in bold indicate the best operating point for each tool for the corresponding metric

We first look at the effect of using Illumina reads along with PacBio reads in the hybrid setting compared to using pure PacBio data with the same PacBio coverage in the standalone setting. For DeepVariant, using 20 × Illumina reads with 15 × PacBio reads in the hybrid setting provides significant reduction in SNV errors, whereas using 20 × Illumina reads with 30 × or 60 × PacBio reads show only small differences. Similarly, HELLO shows significant improvements when 20 × Illumina reads are used along with 15 × PacBio reads, and marginal improvements when 20 × Illumina reads are used with 30 × and 60 × PacBio reads. This indicates that Illumina reads do not add to the quality of SNV calls when PacBio reads are available at sufficient coverage. The performance of both tools for SNV calls compared to standalone Illumina reads is significantly improved when additional PacBio sequencing reads are used in the hybrid setting. But this is an expected outcome given that standalone PacBio SNV performance with 15 × reads outperforms even 50 × SNV results with Illumina reads, even for GATK, which is optimized for short read platforms.

Next, we look at the hybrid setting with added Illumina reads compared to standalone PacBio calling with the same PacBio coverage from the perspective of indel errors. For both DeepVariant and HELLO, using 20 × Illumina sequencing reads in hybrid setting improves indel call errors in all cases by significant margins, except for the case of 60 × PacBio data where performance is very similar with or without Illumina data. Unlike the case of SNVs, PacBio reads are affected by a context-specific agglomeration of indel errors and in these contexts, it may be difficult to find sufficient support for non-erroneous alleles from reads. Meanwhile Illumina reads do not suffer from such issues and hence adding Illumina data seems to be able to neatly complement the PacBio reads in this situation. The indel performances of both tools are also improved with respect to standalone Illumina calls when additional PacBio sequencing reads are used in the hybrid setting.

So far, we have examined how the addition of *extra* PacBio or Illumina reads affects accuracy compared to standalone calling. Then, what if the added reads are not extra, but a *replacement* or a *substitute*? Interestingly, when HELLO is input with 20 × Illumina and 15 × PacBio dataset (total input data coverage of 35x), it outperforms 40 × coverage Illumina sequencing data for indel and SNV calls from all the three methods. In addition, it performs almost as well as 60 × PacBio variant calling using DeepVariant for both indels and SNVs. Coupled with the fact that long reads can provide strong performance for structural variants at 15 × coverage [8], 20 × Illumina with 15 × PacBio hybrid calling may be an interesting use-case for many sequencing experiments where all types of variants (SNVs, indels, and large structural variants) are desired to be called.

Interestingly, prior literature [27] reports an optimal configuration of 25 × coverage PacBio reads with 60 × coverage Illumina reads for hybrid structural variant calling. Newer sequencing techniques from PacBio, such as the CCS read platforms examined in our experiments, can potentially reduce the coverage requirements of both short and long reads in such hybrid setups for structural variant calling. Given that PacBio CCS data can provide accurate structural variants at 15 × coverage, as mentioned above, the recommendation in this article of using 20 × Illumina, and 15 × PacBio CCS reads may also be examined for its potential as a viable candidate for hybrid structural variant calling as well.

**Fig. 3:**
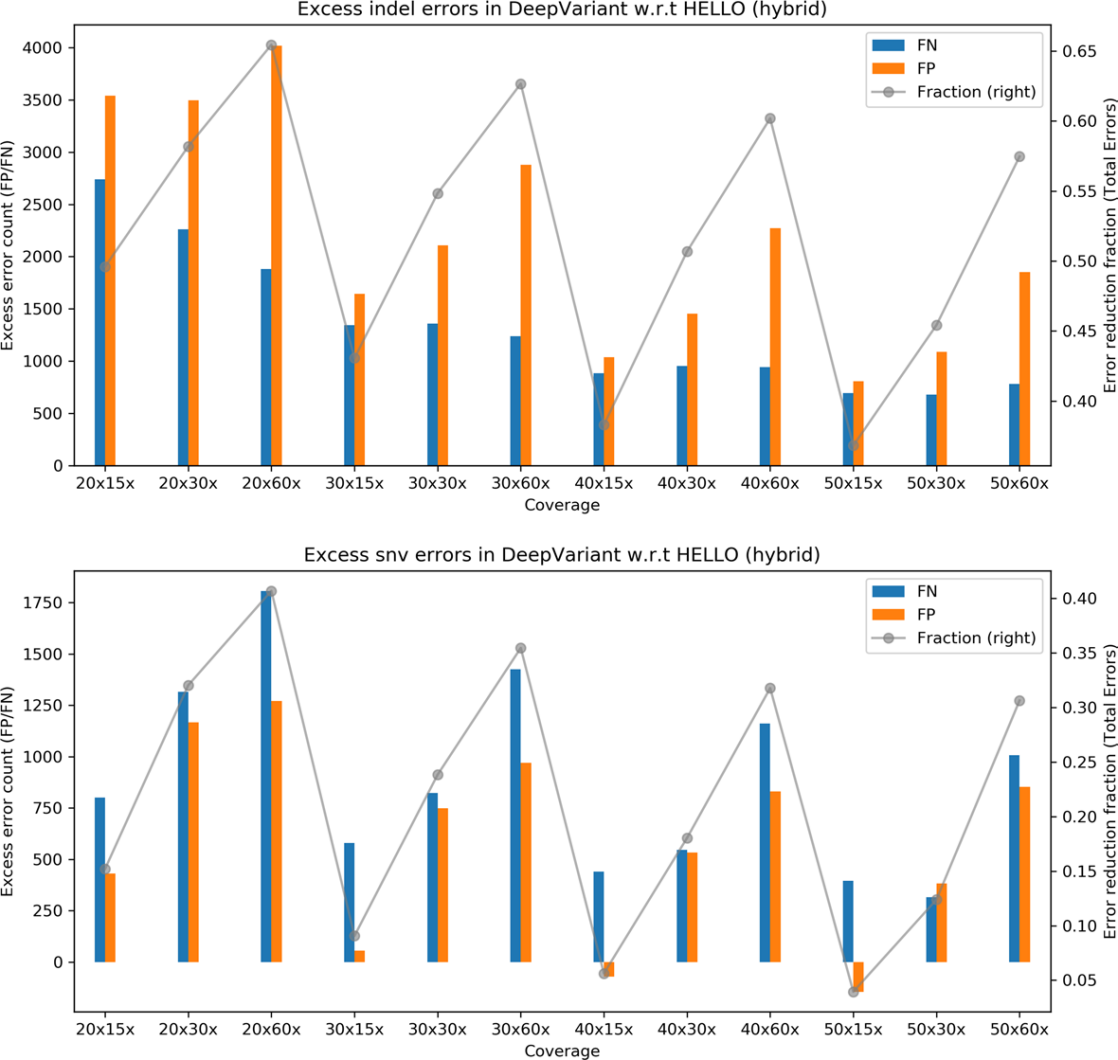
Excess errors in DeepVariant and GATK variant calls compared to HELLO for HG003 hybrid WGS data. Positive values indicate better performance by HELLO over competitor. Bars reference the left y-axis and indicate differences in FP and FN counts between tool (GATK or DeepVariant) and HELLO. The line plots reference the right y-axis, and indicate the differences in total error count as a fraction of total errors of the worse performing method at each coverage point. Please refer to Fig. 1 for additional explanation

To help visualize the relative performance of the methods for the hybrid case, we prepared the plots shown in Fig. 3 that show surplus errors in DeepVariant compared to HELLO. For indel calls, HELLO consistently makes fewer false negative and false positive errors in all cases. Overall, HELLO improves upon the number of erroneous indel calls in DeepVariant by between 40 and 65% approximately. For SNV calls, HELLO makes fewer errors by up to 40% approximately. The advantage held by HELLO decreases at higher Illumina coverages, but increases at higher PacBio coverages for both indels and SNVs.

We examined the number of trainable parameters in DeepVariant’s checkpoints. We found 21,775,363 parameters for both the hybrid and Illumina models, and 21,776,227 parameters for the PacBio model. HELLO uses 1,536,082 parameters for the PacBio model, 1,536,034 parameters for the Illumina model and 2,857,538 parameters for the hybrid model. These numbers indicate that HELLO’s DNNs are 7.6 × to 14.1 × smaller.

#### GIAB HG003 chromosomes 21–22 variant calling

**Fig. 4:**
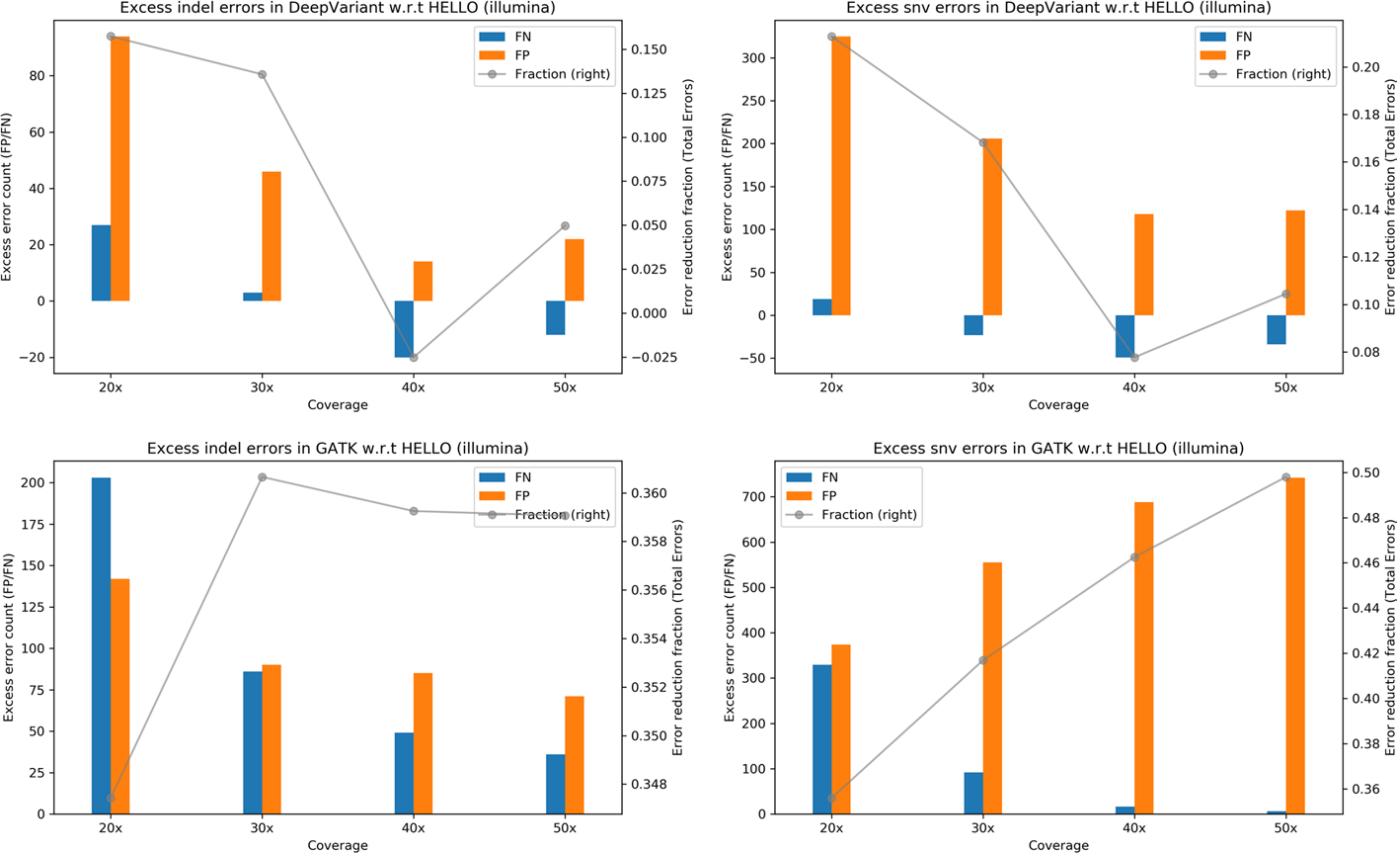
Excess errors in DeepVariant and GATK variant calls compared to HELLO for HG003 Illumina chr21-chr22 data. Positive values indicate better performance by HELLO over competitor. Bars reference the left y-axis and indicate differences in FP and FN counts between tool (GATK or DeepVariant) and HELLO. The line plots reference the right y-axis, and indicate the differences in total error count as a fraction of total errors of the worse performing method at each coverage point. Please refer to Fig. 1 for additional explanation

**Fig. 5:**
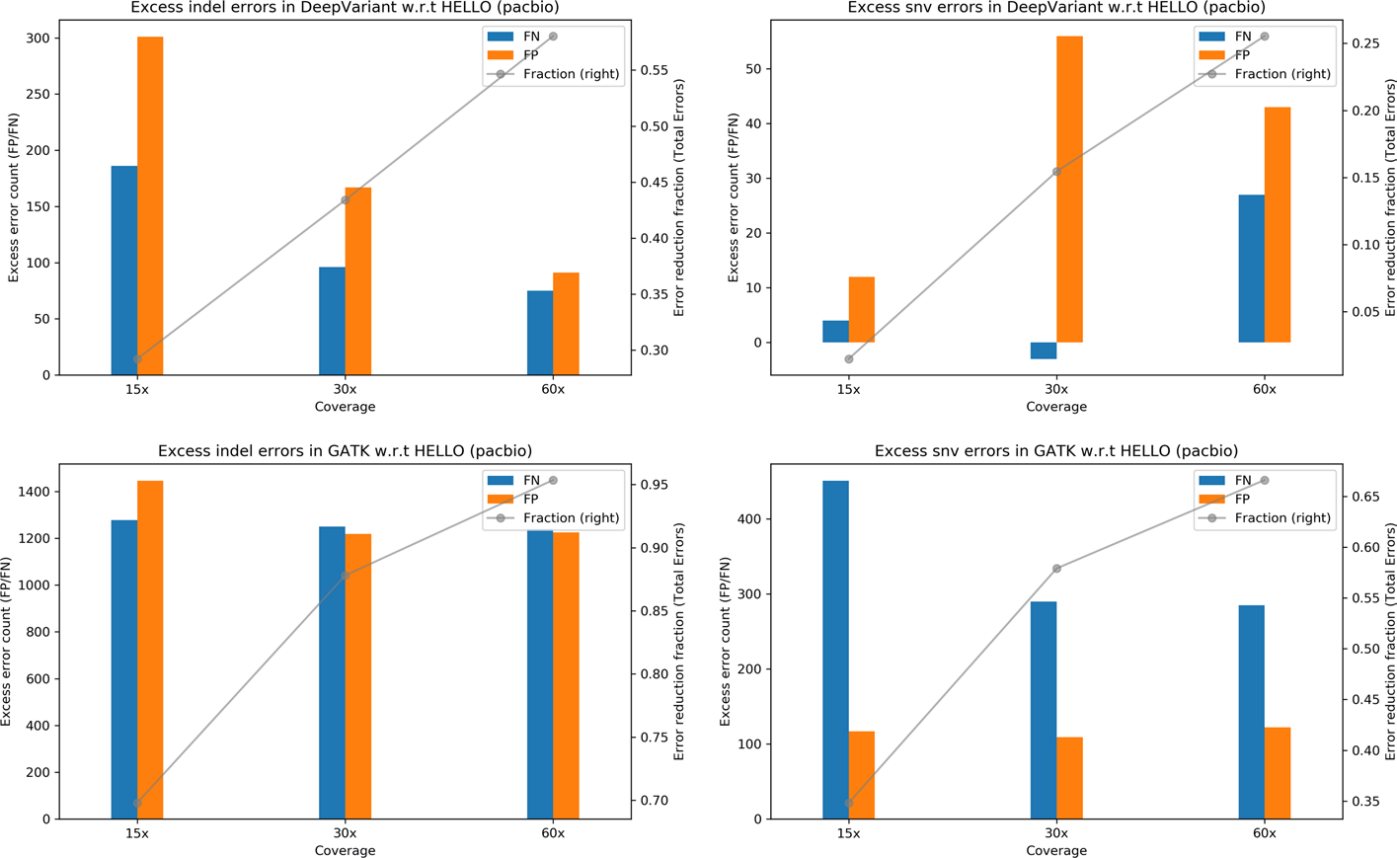
Excess errors in DeepVariant and GATK variant calls compared to HELLO for HG003 PacBio chr21-chr22 data. Positive values indicate better performance by HELLO over competitor. Bars reference the left y-axis and indicate differences in FP and FN counts between tool (GATK or DeepVariant) and HELLO. The line plots reference the right y-axis, and indicate the differences in total error count as a fraction of total errors of the worse performing method at each coverage point. Please refer to Fig. 1 for additional explanation

**Fig. 6:**
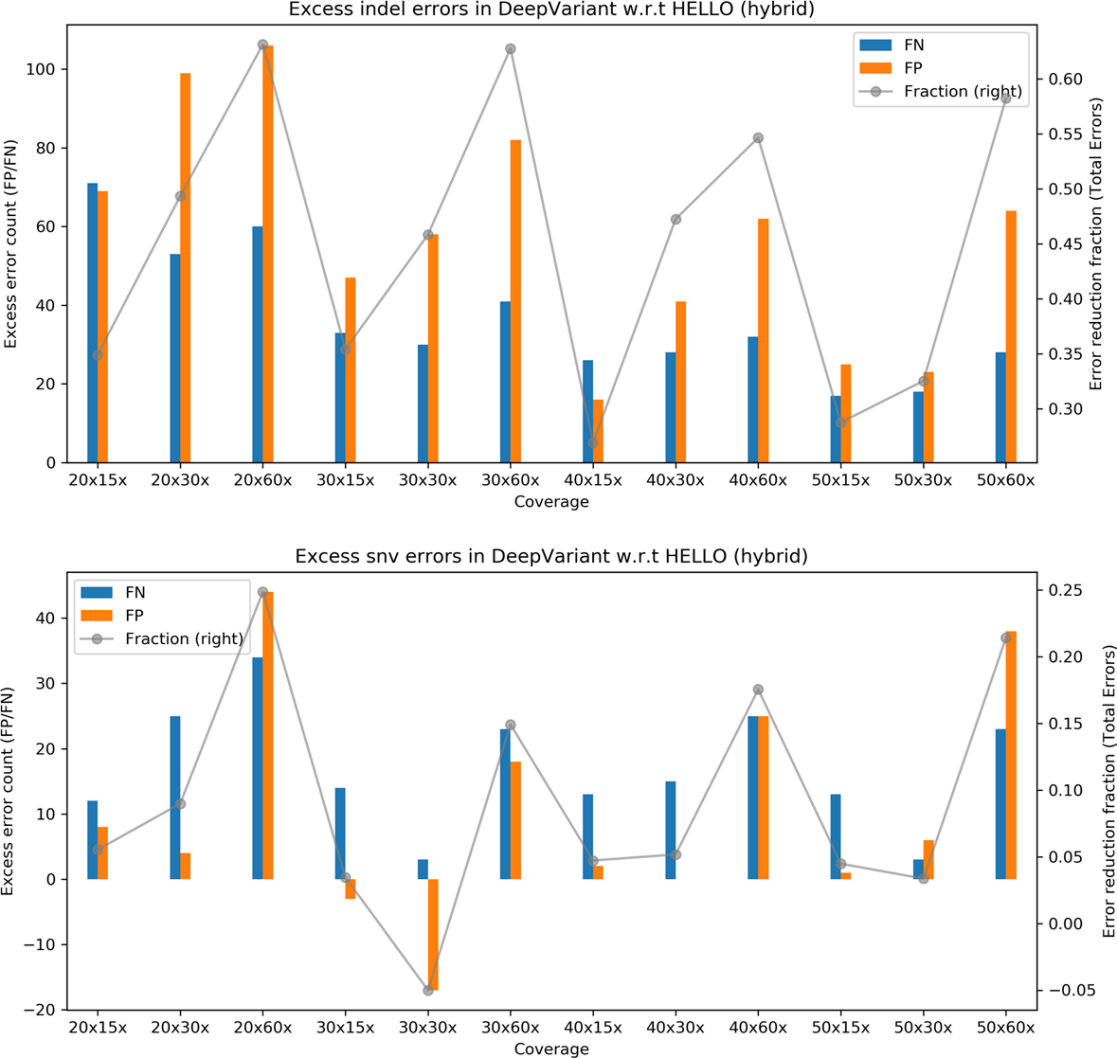
Excess errors in DeepVariant and GATK variant calls compared to HELLO for HG003 hybrid chr21-chr22 data. Positive values indicate better performance by HELLO over competitor. Bars reference the left y-axis and indicate differences in FP and FN counts between tool (GATK or DeepVariant) and HELLO. The line plots reference the right y-axis, and indicate the differences in total error count as a fraction of total errors of the worse performing method at each coverage point. Please refer to Fig. 1 for additional explanation

We examined the variant call performance of the methods for HG003 chromosomes 21 and 22 which were completely held out during training. The results are presented in Tables 7, 8, 9, 10, 11, 12 and Figs. 4, 5, 6. We find that the precisions and recalls for chromosomes 21 and 22 track closely what was observed for the whole genome HG003 calling. In addition, HELLO maintains its lead over competitors in almost all cases.

**Table 7:** Indel results for HG003 chr21-22, Illumina

	GATK			DeepVariant			HELLO		
	Precision	Recall	Errors	Precision	Recall	Errors	Precision	Recall	Errors
20x	0.974686	0.953333	993	0.978402	0.966126	769	0.985003	0.968089	648
30x	0.987363	0.977466	488	0.990523	0.983499	361	0.993741	0.983717	312
40x	0.989344	0.983863	373	0.994353	0.988878	233	0.995318	0.987425	239
50x	**0.991753**	**0.986843**	**298**	**0.995205**	**0.990332**	**201**	**0.996740**	**0.989460**	**191**

Values in bold indicate the best operating point for each tool for the corresponding metric

**Table 8:** SNV results for HG003 chr21-22, Illumina

	GATK			DeepVariant			HELLO		
	Precision	Recall	Errors	Precision	Recall	Errors	Precision	Recall	Errors
20x	**0.991290**	0.985697	1975	0.991890	0.989302	1616	0.995664	0.989523	1272
30x	0.990683	0.991279	1552	0.994724	0.992616	1088	0.997117	0.992349	905
40x	0.989799	0.992535	1522	0.996386	0.993291	887	0.997759	0.992721	818
50x	0.989585	**0.992988**	**1502**	**0.996747**	**0.993453**	**842**	**0.998167**	**0.993058**	**754**

Values in bold indicate the best operating point for each tool for the corresponding metric

**Table 9:** Indel results for HG003 chr21-22, PacBio

	GATK			DeepVariant			HELLO		
	Precision	Recall	Errors	Precision	Recall	Errors	Precision	Recall	Errors
15x	0.862101	0.857818	3903	0.943052	0.937196	1666	0.964158	0.950716	1179
30x	0.902858	0.895181	2812	0.977656	0.979065	606	0.989340	0.986043	343
60x	**0.909926**	**0.904776**	**2585**	**0.990084**	**0.989460**	**286**	**0.996480**	**0.994912**	**120**

Values in bold indicate the best operating point for each tool for the corresponding metric

**Table 10:** SNV results for HG003 chr21-22, PacBio

	GATK			DeepVariant			HELLO		
	Precision	Recall	Errors	Precision	Recall	Errors	Precision	Recall	Errors
15x	0.996704	0.984290	1631	0.997950	0.989488	1079	0.998090	0.989535	1063
30x	0.998283	0.993698	689	0.998906	0.997105	343	0.999558	0.997070	290
60x	**0.998308**	**0.994581**	**611**	**0.999232**	**0.997581**	**274**	**0.999732**	**0.997895**	**204**

Values in bold indicate the best operating point for each tool for the corresponding metric

**Table 11:** Indel results for HG003 chr21-22, Hybrid

	DeepVariant			HELLO		
	Precision	Recall	Errors	Precision	Recall	Errors
20 × 15x	0.986837	0.984444	401	0.991683	0.989605	261
20 × 30x	0.988237	0.989823	308	0.995143	0.993676	156
20 × 60x	0.989732	0.991568	263	0.997114	0.995929	97
30 × 15x	0.992761	0.991059	226	0.996052	0.993458	146
30 × 30x	0.992854	0.993458	192	0.996902	0.995639	104
30 × 60x	0.992244	0.993821	196	**0.997958**	0.996802	73
40 × 15x	0.995501	0.993312	156	0.996622	0.995202	114
40 × 30x	0.994741	0.994839	146	0.997608	0.996874	77
40 × 60x	0.992944	0.994839	172	0.997258	0.997165	78
50 × 15x	0.995572	0.993967	146	0.997323	0.995202	104
50 × 30x	**0.995649**	**0.995348**	**126**	0.997257	0.996656	85
50 × 60x	0.993425	**0.995348**	158	0.997890	**0.997383**	**66**

Values in bold indicate the best operating point for each tool for the corresponding metric

**Table 12:** SNV results for HG003 chr21-22, Hybrid

	DeepVariant			HELLO		
	Precision	Recall	Errors	Precision	Recall	Errors
20 × 15x	0.998487	0.997302	362	0.998581	0.997442	342
20 × 30x	0.998732	0.997512	323	0.998779	0.997802	294
20 × 60x	0.998709	0.997639	314	0.999221	0.998035	236
30 × 15x	0.998894	0.997407	318	0.998860	0.997570	307
30 × 30x	**0.999104**	0.997802	**266**	0.998907	0.997837	280
30 × 60x	0.999011	0.997791	275	0.999221	0.998058	234
40 × 15x	0.998929	0.997372	318	0.998953	0.997523	303
40 × 30x	0.998976	0.997651	290	0.998977	0.997826	275
40 × 60x	0.998953	0.997733	285	0.999244	0.998023	235
50 × 15x	0.999011	0.997349	313	0.999023	0.997500	299
50 × 30x	0.999057	**0.997826**	268	0.999128	0.997860	259
50 × 60x	0.998860	**0.997826**	285	**0.999302**	**0.998093**	**224**

Values in bold indicate the best operating point for each tool for the corresponding metric

The following is a summary of the comparisons.

- For PacBio, HELLO outperforms DeepVariant and GATK in all cases.
- For Hybrid variant calling, HELLO outperforms DeepVariant in all cases except SNV calling for 30 × Illumina and 30 × PacBio reads; DeepVariant makes approximately 5% fewer errors in this case.
- For Illumina variant calling, HELLO outperforms DeepVariant and GATK in all cases except indel calling at 40 × Illumina coverage where DeepVariant makes approximately 3% fewer errors.

#### GIAB HG001 whole genome variant calling

Some of the salient points of the variant calling results for HG001 are reflective of the nature of its ground-truth set and sequencing data, rather than of the variant callers themselves. In addition to having a different ground-truth set as discussed before, GIAB PacBio sequencing data available for HG002 and HG003 are from the newer chemistry 2 process from PacBio whereas for GIAB HG001, the available datasets are from chemistry 1, which indicates that the data quality of PacBio reads may also be poorer in this case. Combined, we expect improvements in Illumina variant call results, a decrease in the accuracy of PacBio results, and smaller differences between the methods in SNV calling for PacBio and hybrid variant calling.

**Fig. 7:**
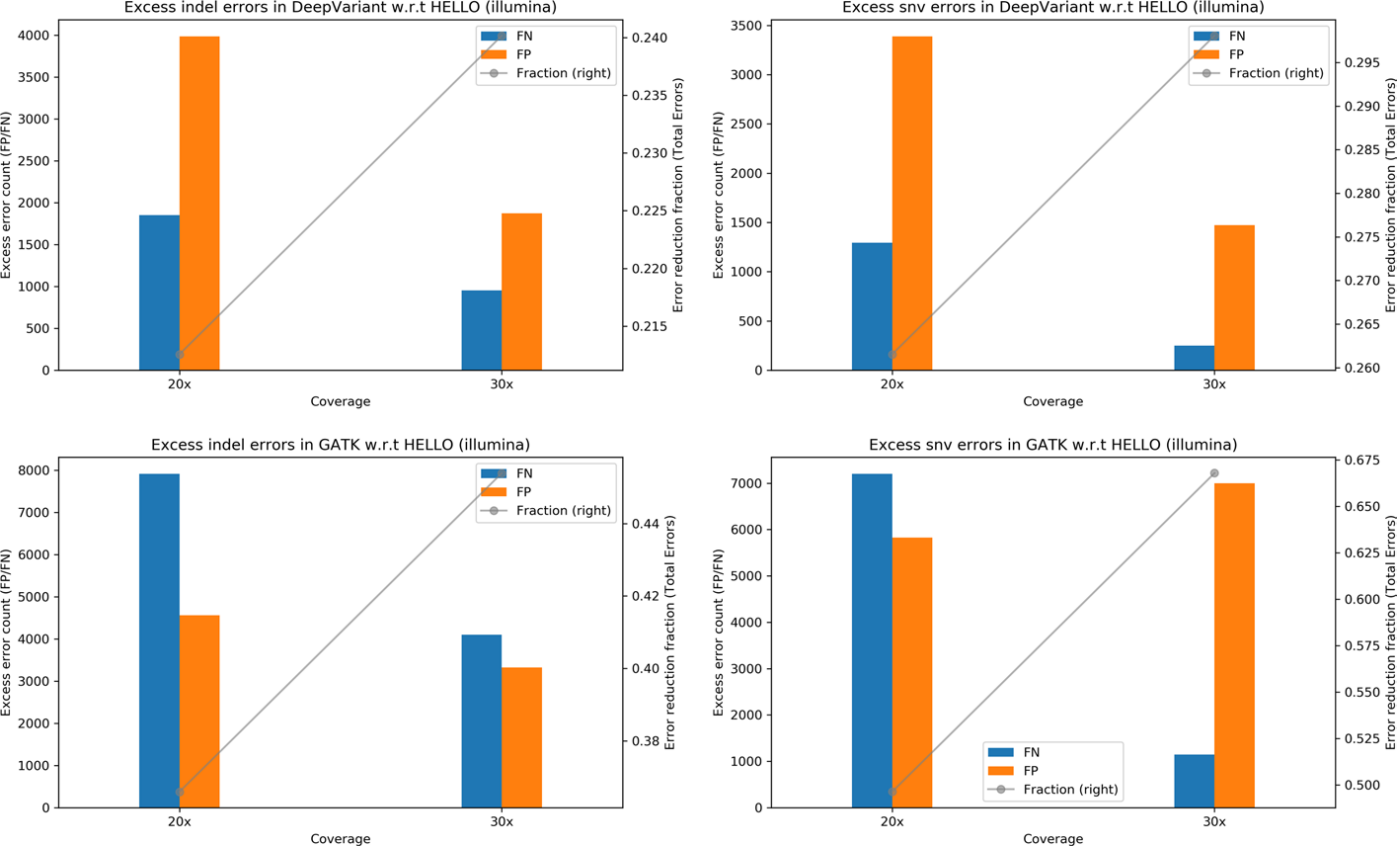
Excess errors in DeepVariant and GATK variant calls compared to HELLO for HG001 Illumina WGS data. Positive values indicate better performance by HELLO over competitor. Bars reference the left y-axis and indicate differences in FP and FN counts between tool (GATK or DeepVariant) and HELLO. The line plots reference the right y-axis, and indicate the differences in total error count as a fraction of total errors of the worse performing method at each coverage point. Please refer to Fig. 1 for additional explanation

**Fig. 8:**
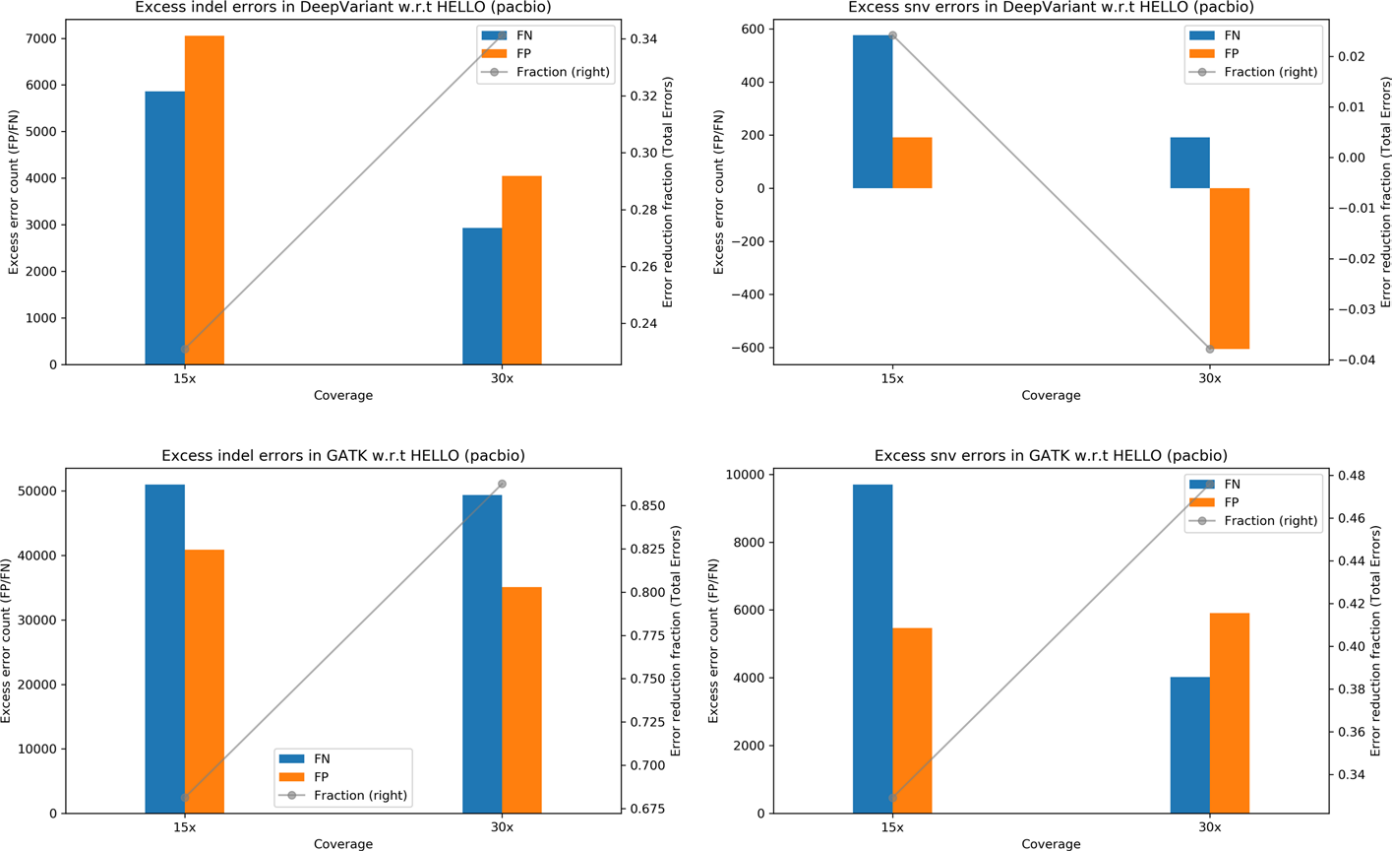
Excess errors in DeepVariant and GATK variant calls compared to HELLO for HG001 PacBio WGS data. Positive values indicate better performance by HELLO over competitor. Bars reference the left y-axis and indicate differences in FP and FN counts between tool (GATK or DeepVariant) and HELLO. The line plots reference the right y-axis, and indicate the differences in total error count as a fraction of total errors of the worse performing method at each coverage point. Please refer to Fig. 1 for additional explanation

**Fig. 9:**
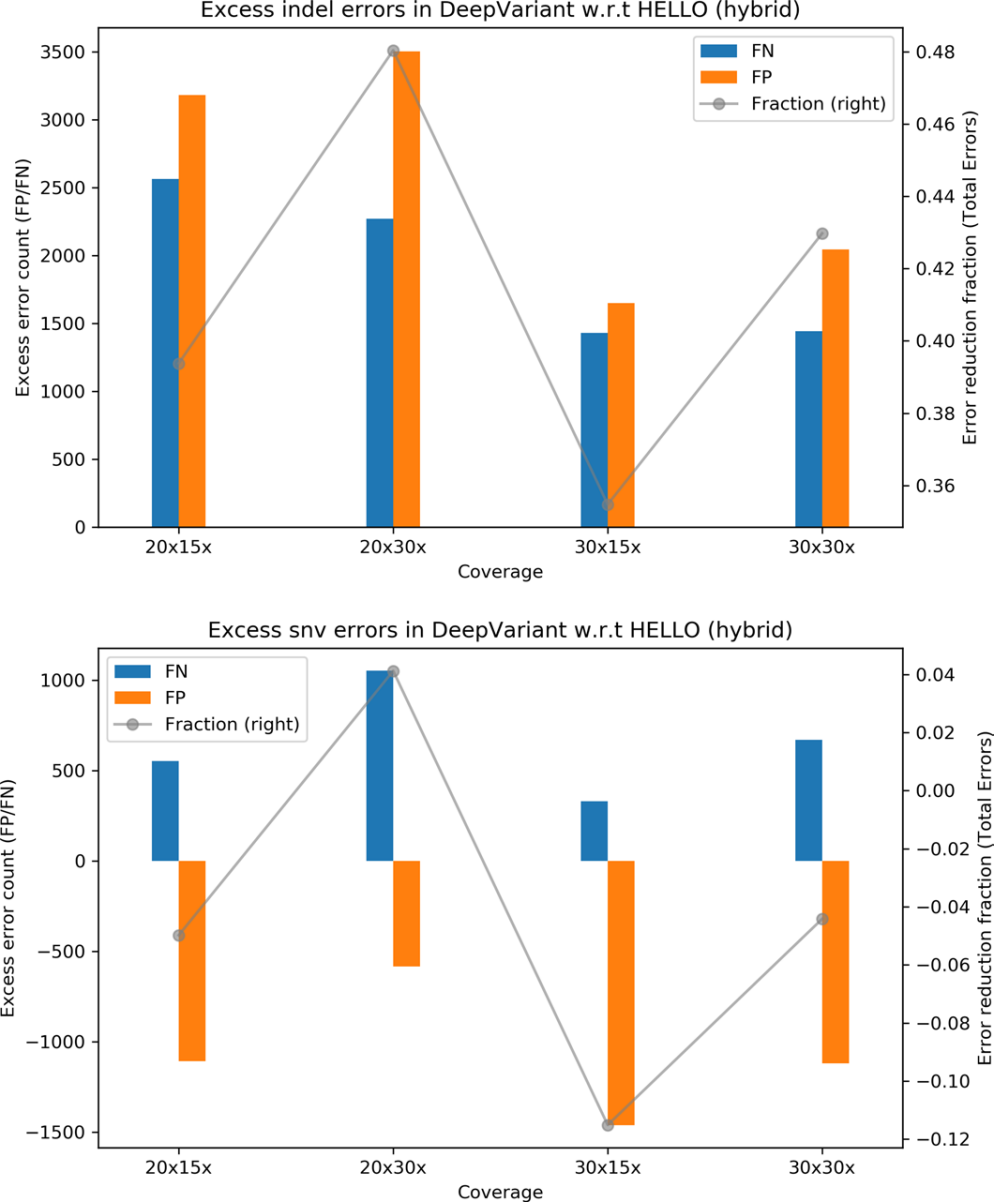
Excess errors in DeepVariant and GATK variant calls compared to HELLO for HG001 hybrid WGS data. Positive values indicate better performance by HELLO over competitor. Bars reference the left y-axis and indicate differences in FP and FN counts between tool (GATK or DeepVariant) and HELLO. The line plots reference the right y-axis, and indicate the differences in total error count as a fraction of total errors of the worse performing method at each coverage point. Please refer to Fig. 1 for additional explanation

Results for whole genome variant calling in HG001 are presented in Tables 13, 14, 15, 16, 17, 18 and relative performances of the tools are presented in Figs. 7, 8, 9. All tools give higher accuracy SNV calls with Illumina rather than PacBio reads for HG001, the opposite situation compared to HG003. In addition, whereas hybrid SNV call performance was better compared to stand-alone Illumina calling in the case of HG003 for both tools at all coverage points, it is not so for HG001. This could be because of a combination of fewer challenging SNV sites for Illumina, and older PacBio sequencing data.

**Table 13:** Indel results for HG001 WGS, Illumina

	GATK			DeepVariant			HELLO		
	Precision	Recall	Errors	Precision	Recall	Errors	Precision	Recall	Errors
20x	0.975429	0.956826	34,098	0.976854	0.968963	27,455	0.984478	0.972669	21,619
30x	**0.986186**	**0.981593**	**16,367**	**0.989031**	**0.987899**	**11,763**	**0.992588**	**0.989808**	**8938**

Values in bold indicate the best operating point for each tool for the corresponding metric

**Table 14:** SNV results for HG001 WGS, Illumina

	GATK			DeepVariant			HELLO		
	Precision	Recall	Errors	Precision	Recall	Errors	Precision	Recall	Errors
20x	0.996457	0.994914	26,242	0.997263	0.996855	17,898	0.998376	0.997280	13,217
30x	**0.997000**	**0.999002**	**12,188**	**0.998809**	**0.999297**	**5767**	**0.999292**	**0.999378**	**4048**

Values in bold indicate the best operating point for each tool for the corresponding metric

**Table 15:** Indel results for HG001 WGS, PacBio

	GATK			DeepVariant			HELLO		
	Precision	Recall	Errors	Precision	Recall	Errors	Precision	Recall	Errors
15x	0.882206	0.848058	134,865	0.951037	0.938379	55,902	0.964688	0.950110	42,985
30x	**0.917539**	**0.887374**	**97,949**	**0.979646**	**0.980324**	**20,445**	**0.987364**	**0.986196**	**13,466**

Values in bold indicate the best operating point for each tool for the corresponding metric

**Table 16:** SNV results for HG001 WGS, PacBio

	GATK			DeepVariant			HELLO		
	Precision	Recall	Errors	Precision	Recall	Errors	Precision	Recall	Errors
15x	0.993747	0.991098	46,067	0.995494	0.994095	31,673	0.995558	0.994285	30,905
30x	**0.994958**	**0.998203**	**20,865**	**0.997091**	**0.999462**	**10,520**	**0.996894**	**0.999524**	**10,934**

Values in bold indicate the best operating point for each tool for the corresponding metric

**Table 17:** Indel results for HG001 WGS, Hybrid

	DeepVariant			HELLO		
	Precision	Recall	Errors	Precision	Recall	Errors
20 × 15x	0.984914	0.986604	14,582	0.990969	0.991731	8840
20 × 30x	0.986462	0.990168	12,013	0.993106	0.994709	6242
30 × 15x	**0.990535**	0.992549	8676	0.993668	0.995411	5598
30 × 30x	0.990507	**0.993720**	**8114**	**0.994388**	**0.996606**	**4627**

Values in bold indicate the best operating point for each tool for the corresponding metric

**Table 18:** SNV results for HG001 WGS, Hybrid

	DeepVariant			HELLO		
	Precision	Recall	Errors	Precision	Recall	Errors
20 × 15x	0.996889	0.999635	10,610	0.996529	0.999816	11,166
20 × 30x	0.996778	0.999513	11,320	0.996589	0.999858	10,853
30 × 15x	**0.997409**	**0.999743**	**8691**	**0.996933**	0.999851	**9822**
30 × 30x	0.997159	0.999649	9738	0.996795	**0.999870**	10,188

Values in bold indicate the best operating point for each tool for the corresponding metric

Comparing the performances of the tools, the following main points may be observed:

For Illumina, HELLO makes approximately 35–45% fewer indel errors compared to GATK and 20–24% fewer indel errors compared to DeepVariant. The improvements are approximately 50–65% and 25–29% respectively for SNVs.

For PacBio, HELLO makes approximately 67–85% fewer indel errors compared to GATK and 22% to 34% fewer indel errors compared to DeepVariant. For SNVs, HELLO makes approximately 32–46% fewer errors compared to GATK. DeepVariant and HELLO perform at par for SNVs with HELLO making 2% fewer errors than DeepVariant at 15x, and DeepVariant making 4% fewer errors than HELLO at 30x.

For hybrid variant calling, HELLO makes approximately 35–48% fewer indel errors than DeepVariant. For SNVs, there is not a single winner with HELLO outperforming DeepVariant by approximately 4% in one case, and DeepVariant outperforming HELLO in other cases with the highest improvement of 12%.

### Other implementation considerations

#### Execution speed, memory consumption and training time

We compared the execution time for HELLO vs DeepVariant on an Intel E5-2683 Central Processing Unit (CPU) for chromosome 21. We used PacBio 30 × coverage data and both models use haplotagged inputs. We used PacBio reads and not Illumina reads so that both pipelines are uniform in terms of input preprocessing steps. HELLO used 28 parallel threads and DeepVariant was configured to use 28 shards (tool option) which may be processed by the tool in parallel. HELLO took 1597 s on average among three runs while DeepVariant took 639 s. We also measured the peak memory and CPU utilization of the two flows over three runs. DeepVariant had a peak CPU utilization of approximately 40 threads and 14.83 GB peak memory usage, while HELLO had a peak CPU utilization of approximately 24 threads and a peak memory usage of 13.04 GB.

HELLO's flow is not optimized for speed and many software components of HELLO may be sub-optimal including reading of sequencing data, preparation of DNN inputs, and the preparation of output files. Improving these components through software engineering practices (for example, removing duplication of work between different stages, better work partitioning so CPU threads are better load-balanced, more efficient conversion of sequencing reads to DNN inputs, etc.) will be part of our future focus.

However, given the smaller DNNs in HELLO, it may be expected that HELLO's DNN runs faster than that of DeepVariant, even when a lot of engineering effort hasn't been spent on optimizing its running time. DeepVariant is divided into three separate scripts—DNN input preparation, DNN inference, and conversion of DNN inference results to variant calls, and the time taken for each stage is reported by DeepVariant. However, the standard HELLO flow is not written into separate scripts, making it difficult to compare the running time of the DNN in HELLO to the running time of the DNN in DeepVariant. To facilitate the comparison, we prepared a new flow for HELLO which operates similarly to the flow in DeepVariant. The new HELLO flow has three scripts as well—the first script prepares input data for the DNN and stores it on disk, the second script reads the DNN inputs, runs them through the DNN and writes the inference results to disk, and the third stage reads the inference results and converts them to output variant calls. It must be noted that this new flow is written for comparing DNN execution times, and for exploratory purposes of HELLO's execution speed and is not the standard HELLO flow. Using this new flow, we compared the execution times of the DNN inference stages of DeepVariant and HELLO on the same compute platform and for the same input data as described above. DeepVariant's DNN takes 338 s on average for completion over three runs, and HELLO takes 273.3 s on average for completion over three runs, indicating that the HELLO DNN is faster than the DeepVariant DNN. For this HELLO flow, we relied on PyTorch to manage the CPU utilization, and we measured a peak CPU utilization of approximately 29 threads and a peak memory usage of 18.28 GB, averaged over three runs. It may be noted that there are various aspects to these execution times and resource utilizations beyond the sole parameter counts of the two DNNs, which could include how input data is prepared for DNN consumption, DNN architectures, how the respective DNN libraries manage machine resources such as memory and CPU usage (PyTorch vs Tensorflow), and others.

Regarding training times, for PacBio data, DeepVariant took approximately 51 min to complete one epoch of training with a batch size of 256 on the Google Cloud TPU v2-8 platform (it has 8 TPUs), and HELLO took approximately 195 min with a batch size of 256 on a Power 9 machine using 2 NVIDIA V100 GPUs. Note that the two compute platforms are very different, and the training times cannot be easily compared. But this comparison provides readers an estimate of how long training the models can take.

### Use of GATK indel realigner

As mentioned before, we used GATK’s indel realigner to preprocess Illumina reads. This step can be avoided by implementing local haplotype assembly and readjustment of read alignments to the most prominent consensus haplotypes within HELLO. Code for doing similar operations is available open source, for example, in DeepVariant’s code repository, which does internal haplotype reassembly to preprocess Illumina reads. However, integrating such code into HELLO will require additional software engineering resources, which we could not allocate so far. We plan to make investments in this direction in the future.

In the meanwhile, it is interesting to measure the impact of not doing indel realignment on the accuracy of HELLO, since avoiding GATK-based preprocessing may be useful for certain users. Towards this goal, we ran variant calling for HG003 chr21 and chr22 without indel realignment, for both Illumina and hybrid variant calling, the two cases which can be influenced by indel realignment. Comparison of these runs to DeepVariant are given in Table 19. HELLO seems to be robust to lack of indel realignment as it continues to perform competitively, outperforming DeepVariant in most of these cases. Note that HELLO models were trained with indel realignment and training the models without indel realignment could help further improve these accuracies.

**Table 19:** HELLO without indel realignment: results for HG003 chr21-22

	DeepVariant			HELLO		
	Precision	Recall	Errors	Precision	Recall	Errors
*a. Illumina indel*						
30x	0.990523	0.983499	361	0.992667	0.981609	356
*b. Illumina SNV*						
30x	0.994724	0.992616	1088	0.997012	0.992349	914
*c. Hybrid indel*						
20 × 15x	0.988237	0.989823	308	0.991680	0.989096	268
*d. Hybrid SNV*						
20 × 15x	0.998732	0.997512	323	0.998581	0.997442	342

## Discussion

A challenge in developing Deep Learning-based methods for variant calling is the enormity of the data required for training, as well as the need for machines with high computational capacity. Models trained for variant calling, even when they are developed for a specific application such as whole-genome calling, need to account for a reasonable spectrum of use-cases such as different coverage points. While popular benchmarks in Deep Learning such as ImageNet do not explicitly check DNN efficacy under different instrument settings (e.g., performance under different levels of lighting for the same set of images), we believe similar checks (e.g., performance against different coverages) are essential in testing variant calling efficacy, since it may be difficult to shoehorn a single variant calling pipeline to operate under a fixed and specific coverage point. Coupled with this, the absence of theoretical guarantees from the Deep Learning machinery doesn’t provide the user confidence that a model works well when targeting different coverages, if such empirical benchmarking is not performed. In addition, variant calling is expected to provide high accuracy solutions compared to many other Deep Learning applications.

Covering any single use case (e.g., coverage point) may require hundreds of Gigabytes of data and hours of training on high-performance hardware. This strategy deviates from traditional approaches which encode domain assumptions necessitating very little, if any, training processes. As genome builds evolve and more difficult-to-call genomic regions become benchmarked, models will need to be trained and improved continually. Methods that perform well under current benchmark regions will need to be reevaluated in the future. Smaller and more efficient models, that have reduced parameter sizes enabled by the introduction of structure and domain assumptions into neural networks, can alleviate some such difficulties. Such models may be more easily trained for cases where procuring large amounts of ground-truth data is difficult or expensive – such as for example in the case of Whole Exome Sequencing, or building models customized to new organisms.

## Conclusions

We presented a novel DNN architecture for small variant calling that encodes the structure of the problem explicitly into the model. Specifically, we model that (i) reads are the fundamental units of sequencing data, (ii) more reads supporting the same candidate allele reinforce the confidence in the allele, and that (iii) the confidence in a candidate allele should be evaluated in relation to other candidate alleles in a simple manner. We introduce strict mathematical relations instead of heuristics to convert model predictions of allelic probabilities into variant calls, and these methods can be used for any ploidy without changing the Neural Network architecture. In experiments, we observe that our model uses 7—14 × fewer parameters than DeepVariant, yet provides variant calls with up to approximately 18 – 65% fewer indel errors across Illumina, PacBio, and hybrid variant calling scenarios.

Structurally well-suited models can provide better solutions to a problem than powerful, general purpose models. To an extent, the demands of *end-to-end* learning from large, monolithic models originally developed for a different, related application such as image recognition, where every aspect of the problem is learnt during training and a few aspects are explicitly coded into the DNN, can be met with very large amount of training data. However, it comes with added costs. Customized models may generalize well (i.e., offer higher accuracy on unseen examples) with a smaller amount of data, and can be advantageous where the correct inductive bias can be coded in by the tool designer.

There are clues in the design of DeepVariant that an image-recognizer may not be the most natural fit for the problem of variant calling, though it is an effective one. These hints manifest in the form of some level of input feature engineering in the DeepVariant pipeline, where (i) for short reads, the reads are sorted and ordered in alignment order and (ii) for PacBio reads, reads are haplotag sorted and aligned to reference and alternative alleles. These feature-engineering steps are aimed at making the input data more well-behaved and more easily accessible to the image processing DNN used in the tool; however, these steps appear arbitrary and the motivation behind them is not well-understood. Our goal is to build a DNN that works for the problem by encoding unmistakable assumptions that can be made about sequencing data and variant calling directly into the structure of the DNN. We also present clear motivations behind these constructions. We hope that these methods lead to improved variant calling accuracy in many use cases, as well as enable future method development for the myriad applications of variant calling.

## Methods

In a typical variant calling pipeline, sequencing data is first aligned to the reference sequence. Alignments indicate where the reads originated from during sequencing. At any site in the reference, we can infer a list of candidate alleles at that site by collecting the list of alleles implied by the reads aligned to that location. Furthermore, we can group the reads aligned to the site by the candidate alleles they imply. A local assembly and realignment of the reads may be performed before enumerating the candidate alleles so that identical alleles are not represented differently by different reads. Insufficient sequencing data can cause the true candidate allele to be missed by all sequencing reads at the site, in which case recovery of the true allele is impossible by looking at the sequencing data.

Traditional methods that probabilistically model sequencing error and read alignment to different candidate alleles have been popular [9]. Here, explicit assumptions and algorithms are used that treat individual sequencing reads as fundamental data units supporting different hypotheses, leading to rules on how to combine support from multiple reads for a candidate allele, and how to evaluate the significance of evidence supporting a candidate allele. For example, one expects a candidate allele with a large proportion of sequencing reads with high base quality and high mapping quality to be a strong candidate, and such notions may be found systematized under this modeling approach.

DeepVariant follows many steps that have been followed traditionally up to a certain point. Local reassembly and realignment are performed for short reads to avoid equivocal representation of variants, and candidate alleles are listed. However, DeepVariant doesn’t explicitly model individual reads and how read support accumulates towards the evidence supporting a candidate allele. Instead pileup data at a site is converted to images with reads supporting different candidate alleles marked or colored differently. This data is then fed into a computer vision DNN, a Convolutional Neural Network (CNN), that predicts, for every non-reference candidate allele and pairs of non-reference candidate alleles, the probabilities for three possibilities – whether the site is homozygous reference, heterozygous, or homozygous alt. These predictions are used to determine scores supporting homozygous/heterozygous calls involving each non-reference candidate allele, and then additional algorithms perform ranking and selection of the top alleles at the site based on these scores. The reason to make predictions in this manner is that a standard CNN makes a fixed number of predictions, and in variant calling the number of candidate alleles and number of true alleles are not fixed. DeepVariant side-steps this discrepancy by decomposing the problem into one where a CNN predicts a fixed number of labels for each subproblem and using an algorithm to post-process these results. HELLO takes a different approach and makes probabilistic arguments to deal with this multi-allelic situation in a precise and well-reasoned manner, as described later.

DeepVariant’s overall approach largely follows the Deep Learning paradigm, in which only a few assumptions are encoded into the mathematical formulation of the problem, and the DNN’s universal function approximation property [28] is leveraged to learn the necessary patterns and rules to make correct predictions by training the model using a large number of training examples. The use of a powerful DNN avoids the use of many, potentially oversimplifying, biases or assumptions, a pitfall of many traditional models. However, it may be possible to encode certain incontrovertible facts or notions about the problem into the Deep Learning machinery that reduces the learning burden of the DNN without affecting the learning efficacy of the algorithms. This approach can lead to more efficient DNN architectures offering higher accuracy solutions.

The DNN architecture in HELLO is structured with DNN elements representing fundamental data units of the problem such as reads and alleles. HELLO recognizes reads as the fundamental units of sequencing data, and the notion that reads that support the same allele reinforce the evidence for that allele. It introduces explicit operations to compare allelic evidence to the data content at a site to determine whether the allelic evidence is significant, or simply represents noise. This comparison recognizes the fact that the support for an allele should be evaluated in relation to the support for the remaining alleles at the site. Finally, HELLO uses simple formulae derived from probabilistic reasoning to produce variant calls from allelic predictions, an approach that can be easily extended to any ploidy without making any changes to the DNN architecture.

### DNN architecture

HELLO uses a DNN to predict the *status* of each candidate allele, the status being either that the candidate allele is a true allele at the site (indicating that the allele is present at the site), or a false allele (indicating that the allele is absent).

HELLO uses different DNN architectures for single-sequencing platform variant calling and dual platform hybrid variant calling. We first describe the single-platform architecture, and then describe how it is modified for the hybrid variant calling case.

### Single-platform architecture

**Fig. 10:**
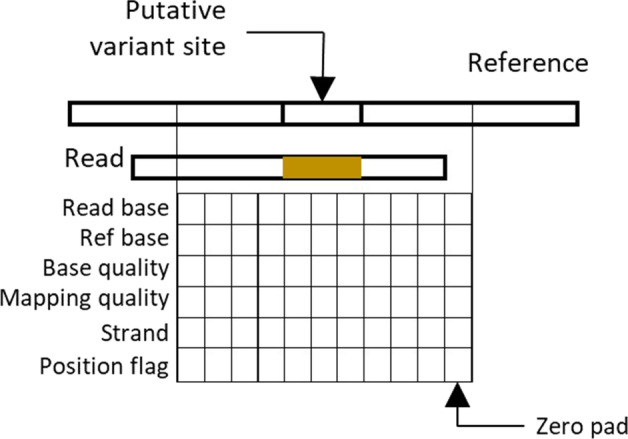
Input read representation

**Fig. 11:**
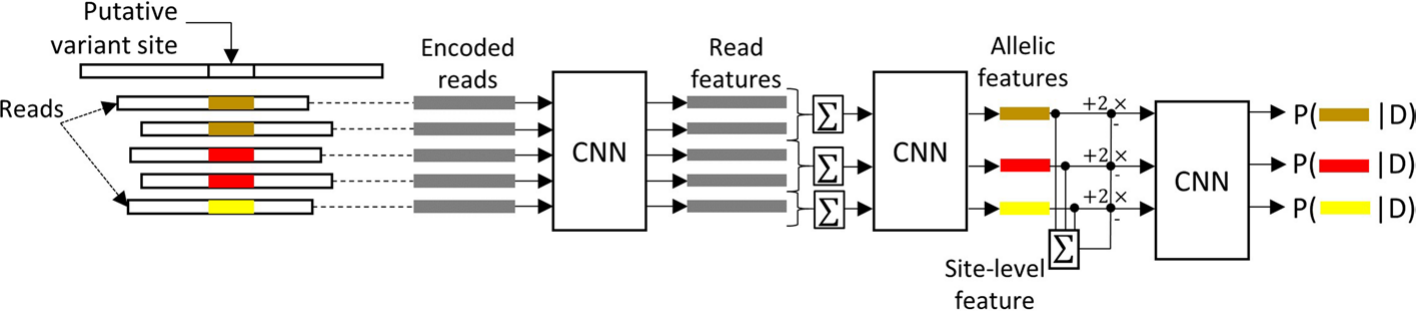
The architecture of HELLO

Figures 10, 11 represents the DNN architecture in HELLO for single sequencing platform analysis. HELLO’s DNN is split into three CNN stages. The input to the first stage is information of read alignments at and around the site being analyzed. How a single read alignment is encoded is indicated in Fig. 10. We collect both the portions of a read aligned to the site being analyzed as well as to surrounding contextual reference locations. The input representation of a read is a sequence of 6-dimensional vectors. Each vector is an encoding of the read base, the reference base, the base quality, the mapping quality of the read, the strand identity of the read, and a position flag indicating whether the vector corresponds to the genomic site that is being analyzed or whether it simply represents a position collected from the surrounding context. For the PacBio haplotag models, we include an additional dimension (not shown in figure) encoding the haplotag value.

Read representations are padded so that the position of the site being analyzed lines up at the center of the representation. The read representations are encoded following DeepVariant’s 8-bit encoding scheme. While it is possible to use floating point numbers or one-hot encoding, or other methods to represent some of these values, the 8-bit representation is compact.

This input representation is processed by the first stage CNN to produce feature representations of reads. The CNN performs 1-D convolutions on individual reads and produces output feature representations for each read, which we call the read-level feature. Read-level features are treated as *superposable*, by which we mean that adding two of these representations is expected to produce a meaningful representation (we depend on the Deep Learning machinery to learn features with such properties). Read feature representations of reads implying the same candidate allele at a site are added together and passed through a second CNN to produce allele-level feature representations. Allele-level feature representations are a summary of the evidence in support of each candidate allele at the site. The final stage CNN compares the allele-level feature for each allele *j* to the allele-level feature of every other candidate allele at the site and outputs the likelihood estimate that allele *j* is a true allele at the site. We further constrain the nature of these comparisons to a specific format that reduces the number of parameters and computations needed. This strategy is described in detail below.

Let $R_{i}$ be the input representation of the *i*th read. Let $f_{i}$ be the read feature representation of the *i*th read. Let $A_{j}$ be the allele-level representation for the *j*th allele, and $S_{j}$ represent the set of reads supporting allele *j*. Let $CNN_{1} , CNN_{2} , CNN_{3}$ be the three CNNs in HELLO. Let $D$ represent the sequencing data at the site including the candidate allele list, and the reads supporting each candidate allele, as well as the reference context. We also define a term, $M = \mathop \sum \limits_{j} A_{j}$, which pools together the evidence from all alleles. $M$ is a representation produced from all the data at a site, thus we call it, the site-level feature. Then, we have the following mathematical relationships for the HELLO architecture.

1 $$f_{i} = CNN_{1} \left( {R_{i} } \right)$$

2 $$A_{j} = CNN_{2} \left(\mathop \sum \limits_{{k \in S_{j} }} f_{k}\right )$$

3 $$P\left[ {allele\, j\, is\, True|D} \right] = p_{j} = CNN_{3} (A_{j} - \mathop \sum \limits_{k \ne j} A_{k} ) = CNN_{3} \left( {2A_{j} - \mathop \sum \limits_{k} A_{k} } \right) = CNN_{3} \left( {2A_{j} - M} \right)$$

Equation 3 indicates the specific type of computations that are used to compare the allelic evidence for one allele to the allelic evidence of all other alleles. That is, an allele’s evidence is judged to be significant only in relation to the evidence presented by all other alleles.

The architecture explicitly defines how alleles are inferred from reads, and how alleles should be compared amongst each other. Enforcing simple mathematical relationships between the basic elements of the problem here induces the relational inductive biases inherent to the problem of variant calling, and limits the DNN to learning feature representations from a subset of feature representations that are meaningful for the problem, rather than a space of possible representations that generally apply to a different class of problems such as, say, image processing problems.

In other words, limiting allelic comparisons to be performed through subtractions in this manner, as well as causing allelic representations to derive from read level representations through superpositions encourages the DNN to search within a solution-space that conforms to certain notions such as (i) more reads supporting a candidate allele result in higher confidence in that candidate allele (ii) the confidence in a candidate allele can be determined only in a *simple relative sense* with respect to the remaining candidate alleles at the site. As a technical aside, note that for $n$ candidate alleles, this requires only $n$ invocations of $CNN_{3}$ instead of $$O\left( {\left( {\begin{array}{*{20}c} n \\ 2 \\ \end{array} } \right)} \right)$$ invocations that would be necessary for a pairwise comparison for all candidates.

After the CNN computations are performed, we have $n$ values, $p_{j} , 1 \le j \le n,$ for $n$ candidate alleles. As indicated in Eq. 3, $p_{j}$ represents the probability of allele *j* being a true allele at the site, which we model to be conditionally independent of the status (true/false) of another allele, given $D$. This gives us the following probabilistic formulation where an outcome, $E$, is an *n-*tuple of values $e_{j} \in \left\{ {True,False} \right\}, 1 \le j \le n$. $e_{j}$ indicates the status of candidate allele *j*. The log-likelihood of the outcome, $E$, is hence given as follows. Here, $1_{v}$ is the indicator function which takes the value 1 when the Boolean variable, $v,$ is $True$, and takes the value $0$ otherwise.

$$\log P\left( {E{|}D} \right) = \mathop \sum \limits_{j = 1}^{n} {\text{log}}(1_{{e_{j} }} p_{j} + 1_{{\overline{e}_{j} }} (1 - p_{j} ))$$

Ploidy of 2 requires the additional assumption that we only look at a subset of the event-space where either one allele has a true status or two alleles have true statuses, and the remaining have false statuses (assuming the true allele is never missed by sequencing reads). Denoting the set of such events as ${\Omega }_{2}$, we have for each $E \in \Omega _{2}$,

$$\log P\left( {E{|}D,ploidy = 2} \right) = \log P\left( {E{|}D} \right) - \log \mathop \sum \limits_{{E^{\prime} \in {\Omega }_{2} }} P\left( {E^{\prime}{|}D} \right)$$

Any event $E \in {\Omega }_{2}$ corresponds to a valid variant call for ploidy = 2. We wish to pick the best event, or the event with the best log-likelihood from this set and determine the corresponding set $V$ of true alleles at the site. This selection may be done based on the following equations.

4 $$E^{*} = argmax_{{E \in {\Omega }_{2} }} {\text{log P}}\left( {E{|}D,ploidy = 2} \right) = argmax_{{E \in {\Omega }_{2} }} \log P(E|D)$$

5 $$V = \{i {|} e_{i}^{*} is True\}$$

Equations 4 and 5 are fairly easy to compute by looping through the set ${\Omega }_{2}$. Once V is determined, HELLO converts it to variant records. If V is a singleton set, HELLO treats the site as homozygous, and if not, it treats the site as heterozygous and prints appropriate VCF records reflecting these determinations.

### Dual-platform (hybrid) architecture

The DNN architecture for calling variants from dual sequencing platforms is derived from the architecture presented earlier for the single sequencing platform. We use two copies of $CNN_{1}$, and $CNN_{2}$ each in the hybrid architecture corresponding to each sequencing platform. CNNs processing Illumina sequencing reads are labeled $CNN_{1}^{I} , CNN_{2}^{I}$ and CNNs processing the PacBio sequencing reads are labeled $CNN_{1}^{P} , CNN_{2}^{P}$. The allelic features for allele *j* output from $CNN_{2}^{P}$ is labeled $A_{j}^{P}$ and the allelic features output from $CNN_{2}^{I}$ is labeled $A_{j}^{I}$. Similarly, the corresponding site-level features are $M^{P}$, and $M^{I}$ respectively. To make allele-level predictions as in Eq. 3, we need to consider allele and site-level features from both platforms. Towards this purpose, we create hybrid versions of both features as follows. Here, $\left( {a,b} \right)$ represents the concatenation operation along the channel dimension.

6 $$A_{j}^{hybrid} = CNN_{4} \left( {\left( {A_{j}^{I} ,A_{j}^{P} } \right)} \right)$$

7 $$M^{hybrid} = CNN_{5} \left( {\left( {M^{I} ,M^{P} } \right)} \right)$$

8 $$p_{j} = CNN_{3} \left( {2A^{hybrid} - M^{hybrid} } \right)$$

$CNN_{4}$ and $CNN_{5}$ are small two-layer networks which take two feature-maps, concatenate them along the channel dimension, and mix them together. We constructed these CNNs in such a manner that the output feature size is the same as the feature size of one of the inputs. Given the hybrid features, we can now rewrite Eq. 3 using hybrid allele and site-level features instead of single-platform features as shown in Eq. 8 and then proceed to perform variant calls using Eqs. 4 and 5.

### Details of DNN construction

The CNNs in HELLO use standalone convolutional layers and Residual Blocks [29] which form the bulk of the model. Residual Blocks use skip connections across multiple convolutional layers. In HELLO, skip connections are used over pairs of convolutional layers. We use rectified linear units as neuronal activations. We apply weight normalization [30] to the DNN layers in HELLO, in favor of the more popular Batch Normalization [31]. Batch Normalization estimates mean and variance statistics of the dataset from running mean and running variance of the layer input batches. In HELLO, the batches may have varying sizes because different sites may have different numbers of reads and candidate alleles because of the nature of sequencing data and hence these estimates may not be sufficiently accurate. Detailed architectural diagrams are provided in the Additional file 1 (Figures S1 – S3).

### Variant calling pipeline

The DNN we described above is embedded into HELLO’s variant calling pipeline which consists of multiple steps. We describe these steps below.

### Indel Realignment for short reads

Short reads aligned independently can represent the same indel deviations from the reference in different ways. In addition, soft clipping can cause some legitimate insertions to be discarded from the aligned section of a read. To circumvent such issues, we realign Illumina reads using GATK 3.8.1’s indel realignment algorithm where reads are locally reassembled and realigned to produce consensus indels. This step is not performed for PacBio reads.

### Determining hotspots

Input alignment files (after realignment) are analyzed to determine for each reference position, the number of read bases in support of the reference bases as well as in support of each non-reference candidate allele. If any non-reference candidate allele has support from a sufficient fraction of reads aligning to the site, the site is determined to be a hotspot and is analyzed using the DNN. For the hybrid variant calling case, we prepare separate hotspots from the two sets of sequencing data from the two sequencing platforms and use the union of the two sets.

### Variant calling

For each hotspot location, we extract all candidate alleles and group the reads according to the candidate alleles they support. We construct input read representations for each read supporting each candidate allele. The input representations are sequences of 1-D sequences of length 150 and dimensionality six (seven for PacBio haplotagged reads) and padded such that the site of interest is at the center of the vectors, as described before. These input representations are fed to HELLO’s DNN architecture and variant calling proceeds according to Eq. 1 to Eq. 8. HELLO uses GNU parallel [32] to launch analysis of multiple hotspots in parallel.

### Preparing training data

Training data preparation for HELLO follows many of the same steps as for variant calling starting with indel realignment and hotspot detection. In the next step, we prepare candidate alleles scanning reads at hotspots, and prepare input data for the DNN. However, since this process is for preparation of data for the training process, the data is not fed into a DNN and instead we have a different step, which is to label the candidate alleles as true or false alleles. To do so, HELLO accepts labeled ground-truth variants and high-confidence regions. Any candidate hotspots outside the high-confidence regions provided as part of the ground-truth set are discarded. Within the high-confidence regions, we label those candidate alleles as true alleles which match the descriptions in the ground-truth variants file. This labeling method is not straight-forward because the allelic representations in the ground-truth variants file may differ from the representation of candidate alleles as inferred by HELLO even when they are identical. To resolve this problem, we group hotspot locations which are nearby and collect all ground-truth variants at or near these locations constructing candidate ground-truth haplotype-pairs for the region. We then search for these haplotype pairs in a prefix-tree induced by the candidate alleles and the surrounding reference segment to locate the true alleles among the candidate alleles inferred by HELLO. The selected candidate alleles are labeled true, and the remaining candidate alleles are labeled false. The details of this algorithm may be seen in our source code and, except perhaps for the prefix-tree-based search implementation, is similar to methods developed elsewhere [10]. The labeled candidate alleles and corresponding read sequence representations are written to disk and are then used to initiate a training process.

## Availability and requirements

**Project name:** HELLO.

**Project homepage:** https://github.com/anands-repo/hello

**Operating system:** Linux.

**Programming Languages:** Python, C +  + 

**Other requirements:** Other software requirements provided in Docker image at https://hub.docker.com/r/oddjobs/hello_image.x86_64

**License:** MIT.

**Restrictions for use by non-academics:** Free to use.

## Supplementary Information

**Additional file 1**. The supplementary file provides details of steps followed in performing experiments, additional results, and additional information regarding the DNN architecture in HELLO

## Data Availability

All experiments in this paper use GIAB data, which are available publicly at ftp://ftp-trace.ncbi.nlm.nih.gov/ReferenceSamples/giab/data. The GRCh38 reference genome is used as standard reference sequence for alignments, and may be found at the following link ftp://ftp.ncbi.nlm.nih.gov/genomes/all/GCA/000/001/405/GCA_000001405.15_GRCh38/seqs_for_alignment_pipelines.ucsc_ids/GCA_000001405.15_GRCh38_no_alt_plus_hs38d1_analysis_set.fna.gz. The ground-truth variants used this paper are downloaded from ftp://ftp.ncbi.nlm.nih.gov/giab/ftp/data/AshkenazimTrio/analysis, ftp://ftp-trace.ncbi.nlm.nih.gov/ReferenceSamples/giab/data/AshkenazimTrio/, and https://ftp-trace.ncbi.nlm.nih.gov/ReferenceSamples/giab/release/NA12878_HG001/, How this data is processed for different variant calling pipelines is described in the Additional file 1 in detail.

## Abbreviations

Indels: Insertions and deletions
NGS: Next generation sequencing
TGS: Third generation sequencing
CCS: Circular consensus sequencing
HMM: Hidden Markov model
DNN: Deep neural network
SNV: Single nucleotide variant
HELLO: Hybrid and stand-alone estimation of small genomic variants
GIAB: Genome-in-a-bottle
GATK: Genome analysis toolkit
TPU: Tensor processing unit
GPU: Graphics processing unit
BAM: Binary alignment map
BWA: Burrows-wheeler aligner
VCF: Variant call format
BQSR: Base quality score recalibration
SNP: Single nucleotide polymorphism
FN: False negative
FP: False positive
CPU: Central processing unit
WGS: Whole genome sequencing
CNN: Convolutional neural network
