# Supplementary material for: HELLO: improved neural network architectures and methodologies for small variant calling
Source: BMC Bioinformatics. 2021 Aug 14;22:404. doi: 10.1186/s12859-021-04311-4 (PMC8364080; doi:10.1186/s12859-021-04311-4)
Supplement: Supplementary file 1 — Additional file 1. The supplementary file provides details of steps followed in performing experiments, additional results, and additional information regarding the DNN architecture in HELLO [file 12859_2021_4311_MOESM1_ESM.pdf]

HELLO: Supplementary Materials

Training data preparation

Illumina sequencing reads

We downloaded 300x Illumina Novoalign BAMS for HG002 from

ftp://ftp-trace.ncbi.nlm.nih.gov/ReferenceSamples/giab/data/AshkenazimTrio/HG002_NA24385_son/NIST_HiSeq_HG002_Homogeneity-10953946/NHGRI_Illumina300X_AJtrio_novoalign_bams/HG002.hs37d5.300x.bam

We converted these BAM files using Picard tools SamToFastq subcommand (with options -I /path/to/HG002.hs37d5.300x.bam -F /path/to/1.fq -F2 /path/to/2.fq -FU /path/to/U.fq).

We then used a python script (available at https://github.com/anands-repo/bamutils/blob/master/split_fastq.py) to split the paired-end fastq files, 1.fq and 2.fq obtained from the step above, into multiple non-overlapping paired-end fastq files through random partitioning. The partitions had the following proportionality of mate-pairs from the source paired-end files: 0.05,0.067,0.083,0.1,0.133,0.167,0.2,0.2 corresponding to 15x, 20x, 25x, 30x, 40x, 50x, 60x, and 60x.

Of these, we performed alignments with the 15x, 20x, 25x, 30x, 40x, and 50x fastq files using BWA mem as follows:

bwa mem -t <NUM_THREADS> $REF /path/to/coverage_point/1.fq /path/to/coverage_point/2.fq -o /path/to/coverage_point/alignment.sam

The reference file, $REF, is downloaded from NCBI at the following link

ftp://ftp.ncbi.nlm.nih.gov/genomes/all/GCA/000/001/405/GCA_000001405.15_GRCh38/seqs_for_alignment_pipelines.ucsc_ids/GCA_000001405.15_GRCh38_no_alt_plus_hs38d1_analysis_set.fna.gz

Read groups were added to the individual alignment files using “samtools addreplacerg” command. We then performed indel realignment for HELLO using GATK Queue version 3.8.1. GATK tool versions prior to GATK version 4 can be found at https://console.cloud.google.com/storage/browser/gatk-software/package-archive/gatk. We prepared a docker image with GATK Queue downloaded from the archive above and Sun Grid-Engine installed for parallel execution. We modified GATK Queue workflow scripts downloaded from https://github.com/UMCUGenetics/GATK-QScripts to work with our setup. Then GATK Queue was launched to run indel realignment as follows

java -jar /path/to/Queue.jar -jobRunner GridEngine -S /path/to/GATK-QScripts/IndelRealigner.scala -R $REF -I $input -mem 10 -nt 24 -nsc 24 -mode "single" -run

Here, $REF is the same reference as above and $input is each alignment file that we prepared

PacBio sequencing reads

PacBio sequencing reads were downloaded from ftp://ftp-trace.ncbi.nlm.nih.gov/ReferenceSamples/giab/data/AshkenazimTrio/HG002_NA24385_son/PacBio_CCS_15kb_20kb_chemistry2/GRCh38

Based on the README file, the alignment files have a coverage of 52x.

Since the aligner used for generating these BAM files, pbmm2, is the same as the aligner we used in our evaluations, we did not perform BAM to fastq followed by alignment for this dataset. Instead, we prepared 8.67x and 17.33x coverage PacBio sequencing reads using our python script written for splitting BAM files into multiple files of different coverages through random partitioning. The partitions had respectively, 1/6th and 1/3rd of the reads in the source BAM file. The script is available here: https://github.com/anands-repo/bamutils/blob/master/split_bams.py

Haplotagging training BAM files

Haplotagging requires a VCF file and a BAM file. To prepare the VCF file for producing haplotagged BAM files for training, we followed the procedure followed by the DeepVariant team (https://github.com/google/deepvariant/issues/369) – which is to use an older version of DeepVariant to prepare variant calls and use these calls to haplotag the BAM files. For this, we first removed haplotags in the alignment files using the following command:

samtools view -h $input | sed -e 's?\t*HP:i:[0-9][0-9]*??g' -e 's?\t*PS:i:[0-9][0-9]*??g' > $UNHAPLOTAGGED

where $input is the input alignment file, and $UNHAPLOTAGGED is the output alignment file without haplotags.

Variant calls were prepared using the following command:

$RUN_DEEPVARIANT_COMMAND --model_type=PACBIO --ref=$REF --reads=$UNHAPLOTAGGED --output_vcf=$dv_vcf_name --num_shards=20

$REF is GRCh38, $dv_vcf_name is the VCF file generated by DeepVariant.

To rehaplotag the reads, we used whatshap to phase the variants and then haplotag the original alignment files as follows:

whatshap phase --output $whatshap_vcf_name --reference $REF $dv_vcf_name $UNHAPLOTAGGED

whatshap haplotag --output $rehaplotagged --reference $REF $whatshap_vcf_name $UNHAPLOTAGGED

We used the same haplotagged BAM file for training the HELLO haplotagged model as well.

Ground truth data

Ground-truth VCF and BED files (referred to as TRUTH_VCF and TRUTH_BED) were downloaded corresponding to the GRCh38 reference corresponding to the HG002 sample from the following link: ftp://ftp.ncbi.nlm.nih.gov/giab/ftp/data/AshkenazimTrio/analysis/NIST_v4.2_SmallVariantDraftBenchmark_07092020

HELLO Illumina training data generation

HELLO is launched for each Illumina BAM file as follows:

python /path/to/hello_dev/python/dump.py --ibam $BAM --ref $REF --truth $TRUTH_VCF --bed $TRUTH_BED --workdir $OUTPUT_DIR --mapq_threshold 5 --num_threads 30 --reconcilement_size 0

HELLO's dump.py only looks at chr1-20 for generating the data, and other reference contigs are ignored. This step produces a list of .memmap, .hdf5 and .index files. The list of .index files can be used as input to the HELLO training script.

DeepVariant Illumina training data generation

DeepVariant requires separate training and validation sets to be provided. We used the following two command templates with DeepVariant v1.1 for generating training and validation sets respectively.

```
seq 0 $((N_SHARDS-1)) | \
   parallel --eta -j $N_SHARDS --joblog ${OUTPUT_DIR}/logs --res ${OUTPUT_DIR}/res \
    /opt/deepvariant/bin/make_examples \
    --mode training \
    --ref "${REF}" \
    --reads "${BAM}" \
    --examples "${OUTPUT_DIR}/training_set.with_label.tfrecord@${N_SHARDS}.gz" \
    --truth_variants "${TRUTH_VCF}" \
    --confident_regions "${TRUTH_BED}" \
    --task {} \
    --regions "'chr1 chr2 chr3 chr4 chr5 chr6 chr7 chr8 chr9 chr10 chr11 chr12 chr13 chr14 chr15 chr16 chr17 chr18'"

seq 0 $((N_SHARDS-1)) | \
   parallel --eta -j $N_SHARDS --joblog ${OUTPUT_DIR}/logs --res ${OUTPUT_DIR}/res \
    /opt/deepvariant/bin/make_examples \
    --mode training \
    --ref "${REF}" \
    --reads "${BAM}" \
    --examples "${OUTPUT_DIR}/validation_set.with_label.tfrecord@${N_SHARDS}.gz" \
    --truth_variants "${TRUTH_VCF}" \
    --confident_regions "${TRUTH_BED}" \
    --task {} \
    --regions "'chr19 chr20'"
```

Shuffling training data

A step needed after training data generation for DeepVariant is shuffling of the data. Here, we describe the procedure used by us for all DeepVariant training runs.

We modified DeepVariant's shuffle_tfrecords_beam.py script to work with Apache Flink (the original script does not work directly as it is written for Google Dataflo). Our modified script is available at https://github.com/anands-repo/deepvariant/blob/r1.0/tools/shuffle_tfrecords_beam.py. For running

this locally, we setup an Apache Flink cluster using docker containers running on multiple nodes. Then the shuffle script is launched as follows.

```
$SHUFFLE_SCRIPT \
        --input_pattern_list="$INPUT_PATTERN" \
        -- output_pattern_prefix=$OUTPUT_PATTERN \
        --output_dataset_config_pbtxt=$OUTPUT_DATASET_CONFIG_PBTXT \
        --output_dataset_name="$OUTPUT_DATASET_NAME" \
        --runner="FlinkRunner" \
        --flink_master="$FLINK_MASTER" \
        --environment_type=PROCESS \
        --environment_config='{"command": "/opt/tools/boot"}' \
        --parallelism $PARALLELISM
```

For commands to run shuffle on Google cloud, please refer to Google's documentation. The environment variables used here are self-explanatory. The output config file $OUTPUT_DATASET_CONFIG_PBTXT is used as an input to the DeepVariant training script. In the end, we shuffled Illumina data on Google Cloud and PacBio and hybrid data on our local clusters.

DeepVariant PacBio training data generation

For PacBio training data generation, we added the following options to the command template for make_examples that was presented for the Illumina case: --norealign_reads --vsc_min_fraction_indels 0.12 --alt_aligned_pileup 'diff_channels' --add_hp_channel --sort_by_haplotypes --parse_sam_aux_fields. This is based on an examination of run_deepvariant.py where for PacBio sequencing reads, the above additional options are used for PacBio variant calling with haplotagged data.

HELLO PacBio training data generation

HELLO is run using a similar command as for Illumina to generate PacBio training examples, only the files are passed using --pbam instead of --ibam. For the model that uses haplotagged information, we pass the additional '--include_hp' option. We use the same BAM file as for DeepVariant training in this case.

HELLO hybrid training data generation

For hybrid calling, we create random combinations of Illumina and Pacbio BAM files for each chromosome. The following is the simple algorithm used for this purpose. Let **illumina_bam_files** be the set of Illumina BAM files, and **pacbio_bam_files** be the set of PacBio BAM files, then

For *chrom* in [1, 20]:
    For *ibam* in **illumina_bam_files**:
        pbam = randomly select from **pacbio_bam_files**
        Create HELLO training data using *ibam*, *pbam* for chromosome *chrom*

The command template below is used to generate the training data for a single chromosome

```
python /path/to/hello_dev/python/dump.py --ibam $ibam --pbam $pbam -ref $REF --workdir $OUTPUT_DIRECTORY --truth $TRUTH_VCF --bed $TRUTH_BED --hybrid_eval --no_data_lst --q_threshold 10 --mapq_threshold 5 --num_threads 40 --reconcilement_size 0 --chromosomes $chrom
```

As may be seen, HELLO accepts two BAM files and a chromosome designation.

DeepVariant hybrid training data generation

We collect the {*chrom*, *ibam*, *pbam*} combinations generated by the above algorithm. We create a merged *ibam+pbam* BAM file for the corresponding *chrom*. We then overwrite read group information to be the same as needed by DeepVariant for hybrid variant calling. The following command templates are used:

samtools view -b -@ 30 $pbam $chrom > $pbam_ part

samtools sort -@ 30 $pbam_ part > $pbam_ part_sorted

samtools view -b -@ 30 $ibam $chrom > $ibam_part

samtools sort -@ 30 $ibam_part > $ibam_part_sorted

samtools merge $merged_bam_part $pbam_part_sorted $ibam_part_sorted

java -jar /path/to/picard.jar AddOrReplaceReadGroups --INPUT $merged_bam_part --OUTPUT $merged_bam_part_RG --RGLB GIAB_Prep --RGPL HYBRID --RGPU unit1 --RGSM SAMPLE01

We invoke make_examples for each $merged_bam_part_RG with options appropriately set. We keep all tfrecords produced for chr1-chr18 for training and those produced for chr19 and chr20 separate for validation.

Training procedure

For training HELLO, we collect all ".index" files produced by the dump.py script runs and list them in a data.lst file. We also need to specify the architecture of the DNN that is being used. The command template is as follows:

```
python /path/to/hello_dev/python/MixtureOfExpertsDNNFast.py \
   --data /path/to/data.lst \
   --config $CONFIG \
   --numWorkers 10 \
   --numEpochs $NUM_EPOCHS \
   --numEarly $NUM_EPOCHS \
   --batchSize $BATCH_SIZE \
   --cuda \
   --lr 3e-4 \
   --outputPrefix $OUTPUT_DIR/$NAME \
   --optimizer Adam \
   --checkpointArchive $OUTPUT_DIR/checkpoint \
   --useMultiGPU \
   --moeType attention \
   --tensorLog $OUTPUT_DIR/${NAME}_tensorlog \
   --binaryClassifier \
   --maxReadsPerSite $MAX_READS_PER_SITE
```

As may be noted the learning rate used is 0.0003 and is used uniformly for training all models. $CONFIG is the name of a python module that is inside HELLO's codebase and describes different model

architectures. HELLO selects 10% of training examples as the validation set. After each training epoch, HELLO evaluates the training loss on the validation set and writes the model configuration to disk if the validation loss has improved over the previous best. The training was performed on IBM POWER8 machines with 4 K80 GPUs each or an IBM POWER9 machine with 2 V100 GPUs.

For the PacBio haplotag models, due to time constraints, we followed a different method. We prepared a script to run PyTorch's distributed data-parallel training procedure which allowed us to use 12 K80 GPUs simultaneously to train the model. This script (MixtureOfExpertsDNNFastDistributed.py) doesn't perform validation in each epoch. Hence, we used chromosomes 1-18 for training and performed validation outside the training loop using chromosomes 19 and 20. This validation may be performed using the MixtureOfExpertsDNNFast.py script with an option --onlyEval passed to it. The value of MAX_READS_PER_SITE was set to 200 for all models except the PacBio haplotag model, for which we set it to 100.

For training DeepVariant, we collect the output config files produced from the shuffle scripts and use the following command template to run training.

```
/opt/deepvariant/bin/model_train \
        --use_tpu \
        --master="grpc://<TPU_IP_ADDR>:8470" \
        --dataset_config_pbtxt=${TRAINING_SET_PBTXT} \
        --train_dir=${TRAINING_RESULTS} \
        --model_name="inception_v3" \
        --number_of_steps=$TOTAL_NUM_BATCHES \
        --save_interval_secs=-1 \
        --save_interval_steps=$NUM_BATCHES_PER_EPOCH \
        --batch_size=$BATCH_SIZE \
        --learning_rate=0.0005 \
        --start_from_checkpoint=""
```

The learning rate used for DeepVariant is 0.0005. This is based on the DeepVariant training tutorial given in the DeepVariant github repository. We used Google Cloud TPU v2 to train these models as DeepVariant builds aren't available for IBM machines and our clusters have GPUs only on IBM machines.

DeepVariant needs another script to be run to perform validation. This script coordinates with the training script and is usually launched during the training process itself. We performed validation after all training checkpoints were dumped from DeepVariant and this required writing a shell script to coordinate with DeepVariant's validation script. The shell script is available at https://github.com/anands-repo/deepvariant/blob/r1.0/tools/post_eval.sh. The command template for running DeepVariant's validation script is as follows

```
python $MODEL_EVAL_PATH \
--dataset_config_pbtxt=$VALIDATION_SET_PBTXT \
--checkpoint_dir="$EVALUATION_DIRECTORY" \
--batch_size=$BATCH_SIZE
```

Simultaneously we launch "/path/to/post_eval.sh $TRAINING_DIRECTORY $EVALUATION_DIRECTORY" to coordinate evaluation with the model_eval script.

Both HELLO and DeepVariant were trained for 20 epochs. In cases where the final epoch produced the best validation accuracy, we trained additional iterations starting from the last checkpoint of the previous training cycle, launched for 5 additional epochs first, then for three until we found that the final epoch's accuracy had degraded compared to a previous one. This had to be done only for the case of DeepVariant's hybrid model and DeepVariant's hybrid model was trained for 28 epochs whereas all other HELLO and DeepVariant models were trained for 20 epochs each.

Test data preparation

Illumina datasets

We downloaded 300x alignment files from ftp://ftp-trace.ncbi.nlm.nih.gov/ReferenceSamples/giab/data/AshkenazimTrio/HG003_NA24149_father/NIST_HiSeq_HG003_Homogeneity-12389378/NHGRI_Illumina300X_AJtrio_novoalign_bams/HG003.GRCh38.300x.bam

For HG001, we obtained 300x BAM files from ftp://ftp-trace.ncbi.nlm.nih.gov/ReferenceSamples/giab/data/NA12878/NIST_NA12878_HG001_HiSeq_300x/NHGRI_Illumina300X_novoalign_bams/HG001.hs37d5.300x.bam

To obtain 20x, 30x, 40x, and 50x BAM Files, we subsampled a 300x BAM file at fractions of 0.067, 0.1, 0.133, and 0.167. Command template is

```
samtools view -b -s ${seed}.${split_proportion} -@ 30 $bam_source > $bam_target
```

$seed is generated using bash $RANDOM functionality. We produced only 20x and 30x BAM files for HG001.

Each BAM file was converted to FASTQ file using Picard SamToFastq subcommand, and then aligned to the GRCh38 reference using bwa mem using the following command template.

```
bwa mem -t 30 $REF /path/to/1.fq /path/to/2.fq -o /path/to/alignment.sam
```

Following methods previously described we performed indel realignment for HELLO. For GATK, we performed BQSR using the following command template

```
gatk BaseRecalibrator -I $INPUT_BAM -R $REF \
   --known-sites $resources_dir/Homo_sapiens_assembly38.dbsnp138.vcf \
   --known-sites $resources_dir/Homo_sapiens_assembly38.known_indels.vcf.gz \
   --known-sites $resources_dir/Mills_and_1000G_gold_standard.indels.hg38.vcf.gz \
   -O $outputdir/recal_data.table

gatk ApplyBQSR \
   -R $REF \
   -I $INPUT_BAM \
   --bqsr-recal-file $outputdir/recal_data.table \
   -O $OUTPUT_BAM
```

The resource files needed were downloaded from the Google Cloud bucket, gs://gcp-public-data--broad-references/hg38/v0/

PacBio datasets

Source BAM file for HG003 was downloaded from ftp://ftp-trace.ncbi.nlm.nih.gov/ReferenceSamples/giab/data/AshkenazimTrio/HG003_NA24149_father/PacBio_CCS_15kb_20kb_chemistry2

For HG001, the source BAM is from wget ftp://ftp-trace.ncbi.nlm.nih.gov/ReferenceSamples/giab/data/NA12878/PacBio_SequelII_CCS_11kb/HG001.SequelII.pbmm2.hs37d5.whatshap.haplotag.RTG.trio.bam

Let's call the downloaded bam file for HG003, $dbam.

We performed BAM to FASTQ conversion using Picard tools, and we aligned the reads to the GRCh38 reference sequence using pbmm2 using the following command template.

```
java -jar /path/to/picard.jar SamToFastq -I $dbam -F /path/to/reads.fq
```

```
pbmm2 align -j 20 $REF /path/to/reads.fq /path/to/$abam --preset CCS –sort –rg '@RG\tID:HG003\tSM:SAMPLE1\tPL:PacBioCCS'
```

The realigned file, as may be seen, is termed $abam. Note that $abam and $dbam have the same coverage.

We subsampled $dbam to a fraction 0.5 using samtools view, and realigned the reads using Picard SamToFastq followed by the same pbmm2 command template above.

We subsampled $abam to a fraction 0.25 using samtools view and realigned the reads to GRCh38 reference using Picard SamToFastq followed by the same pbmm2 command above. This subsampling could equally be done with $dbam, however, we had removed $dbam due to lack of disk space.

This procedure gives us 15x, 30x, and 60x reads. This is because $dbam has a higher sequencing depth than the PacBio training set BAM, however we could not find the coverage information in the README. To determine the sequencing depth of the test set BAM, we counted the number of bases in $abam as well as the training set BAM file for PacBio and used the ratio. That is

$$cov\_test = cov\_train * \#bases(test)/\#bases(train)$$

Setting cov_train to 52x based on the associated README for the training set, cov_test comes out to be 60x.

To count the number of bases in a BAM file, we used the following command

```
samtools bam2fq $1 | awk 'NR%4==2' | awk '{ print length($0) }' | awk '{s += $1} END {print s}'
```

For HG001, we performed similar steps to prepare 15x and 30x coverage reads and note that HG001 is mentioned to be 30x coverage in the README released with he data.

Haplotagging PacBio BAMs for variant calling

As mentioned in the main draft, we train two models for HELLO – one with haplotagging and the other without. We used the HELLO's non-haplotagged model to call variants on the PacBio BAM files first. Then we used those variant calls with Whatshap to produce haplotagged BAM files.

The Whatshap invocations are similar to that presented before except that for 15x coverage data for HG001 and 30x coverage data for HG003 we had to use the option ` --distrust-genotypes` to allow the phasing step the freedom to discard certain heterozygous variant records in the vcf file.

We used the same haplotagged BAM files for variant calling with HELLO and DeepVariant.

Hybrid datasets

HELLO accepts two BAM files one for Illumina and one for PacBio, so no additional BAM file is needed. For DeepVariant, we performed "samtools merge" to produce merged versions of BAM files for each of the hybrid experiments. We used Picard to apply the same read group on all the reads using the following command template.

```
java -jar /path/to/picard.jar AddOrReplaceReadGroups --INPUT $MERGED_BAM --OUTPUT
$MERGED_RG_BAM --RGLB "GIAB_library" --RGPL "HYBRID" --RGPU unit1 --RGSM SAMPLE1
```

Variant calling

Illumina datasets

HELLO command template:

```
python /path/to/hello_dev/python/call.py --ibam $bam --ref $REF --network $network --num_threads
$NUM_THREADS --workdir $workdir --mapq_threshold 5
```

DeepVariant command template:

```
/opt/deepvariant/bin/run_deepvariant \
  --model_type=WGS \
  --customized_model=$BEST_CHECKPOINT \
  --ref=$REF \
  --reads=$bam \
  --output_vcf=$WORKDIR/dv.vcf \
  --num_shards=$NPROCS
```

GATK command template:

1. HaplotypeCaller is run for each chromosome

   ```
   gatk HaplotypeCaller --pcr-indel-model NONE -R $REF -I $bam -O
   $workdir/gatk_chromosome${chrom}.vcf -L chr${chrom}
   ```

2. The variants from the above are all merged and varant scoring is run for the result

   ```
   gatk CNNScoreVariants -V $merged_vcf -R $REF -O $scored_vcf -I $bam -tensor-type
   read_tensor
   ```

3. Filter variants is applied on the scored VCF to obtain the final vcf

   gatk FilterVariantTranches -V $scored_vcf $resources_option --info-key CNN_2D --invalidate-previous-filters -O $filtered_vcf

   $resources_option refers to dBSNP and known indel files that we referenced before downloaded from the GATK resource bundle. $resources_option is of the format "--resources …"

PacBio datasets

HELLO's command template is as follows. The --ibam option used for Illumina is changed to --pbam. For haplotagged BAM files, the option--include_hp is added

```
python /path/to/hello_dev/python/call.py \
   --pbam $BAM \
   --ref $REF \
   --network $network \
   --num_threads 30 \
   --workdir $workdir \
   --mapq_threshold 5
```

DeepVariant command template is

```
/path/to/deepvariant/bin/run_deepvariant \
  --model_type=PACBIO \
  --customized_model=$BEST_CHECKPOINT \
  --ref=$REF \
  --reads=$BAM \
  --use_hp_information \
  --output_vcf=$WORKDIR/dv.vcf \
  --num_shards=$NPROCS
```

GATK command template is based on https://www.nature.com/articles/s41587-019-0217-9, and run as follows

1. HaplotypeCaller was run using --pcr-indel-mode AGGRESSIVE, with minimum mapping quality setting of 60, and -ERC option set to GVCF.
2. GVCF to VCF conversion using scripts released at https://github.com/PacificBiosciences/hg002-ccs/
3. VCF filtration using scripts released at https://github.com/PacificBiosciences/hg002-ccs/

Hybrid datasets

HELLO's command template is as follows where it accepts both --ibam and --pbam options. The option reconcilement_size should be set to 0 as of now, which disables the reconcilement feature. This is an untested feature that tries to reconcile differing representations of identical alleles by Illumina and PacBio aligners. Currently the method is not tuned properly and can take a large amount of time to run.

```
python /path/to/hello_dev/python/call.py \
```

```
  --ibam $ibam \
  --pbam $pbam \
  --ref $ref \
  --network $network \
  --num_threads 30 \
  --workdir $workdir \
  --mapq_threshold 5 \
  --reconcilement_size 0
```

DeepVariant's command template is as follows. The input BAM file in this case is the merged, read-group imputed BAM files we described before.

```
/path/to/deepvariant/bin/run_deepvariant \
  --model_type=HYBRID_PACBIO_ILLUMINA \
  --customized_model=$model \
  --ref=$REF \
  --reads=$workdir/merged.RG.bam \
  --output_vcf=$dv_vcf_name \
  --num_shards=20"
```

Evaluating results

We downloaded the following ground-truth VCF and BED files for HG003 corresponding to GRCh38:

https://ftp-trace.ncbi.nlm.nih.gov/ReferenceSamples/giab/release/AshkenazimTrio/HG003_NA24149_father/latest/GRCh38/HG003_GRCh38_1_22_v4.2.1_benchmark.vcf.gz

https://ftp-trace.ncbi.nlm.nih.gov/ReferenceSamples/giab/release/AshkenazimTrio/HG003_NA24149_father/latest/GRCh38/HG003_GRCh38_1_22_v4.2.1_benchmark_noinconsistent.bed

Truth set for HG001 was downloaded from https://ftp-trace.ncbi.nlm.nih.gov/ReferenceSamples/giab/release/NA12878_HG001/NISTv3.3.2/GRCh38/

We used hap.py evaluation tool with the vcfeval engine using the following command template using the docker image at pkrusche/hap.py

```
/path/to/hap.py/bin/hap.py \
  $TRUTH_VCF \
  $result_vcf \
  -f $TRUTH_BED \
  -r $REF \
  --threads 10 \
  --engine vcfeval \
  -o ${filename}_eval
```

For DeepVariant when evaluating pacbio output vcfs, hap.py crashed. We found the failure documented in the issue at hap.py github repository at https://github.com/Illumina/hap.py/issues/37 and followed the workaround suggested there to filter out homozygous reference calls, which are not used by hap.py.

Tool versions

The versions of the major tools used are as follows

1. DeepVariant: 1.1 (first-party docker image)
2. GATK: 4.2.0.0 (first-party docker image)
3. samtools: 1.10 (built from source)
4. Picard tools: 2.23 (first-party github release)
5. BWA: 0.7.17-r1198-dirty (built from source)
6. pbmm2: 1.3.0 (conda install)
7. GATK Queue (for Indel realignment): 3.8.1 (from Broad Institute gcloud archives: https://console.cloud.google.com/storage/browser/gatk-software/package-archive, login needed).
8. Whatshap: 1.0 (pip install)

HELLO vs HELLO without haplotagging for PacBio data

Table 1. HELLO vs HELLO without haplotagged data, comparisons for HG003 WGS, PacBio

a) Indel errors

	HELLO			HELLO (NO HP)		
	**Precision**	**Recall**	**Errors**	**Precision**	**Recall**	**Errors**
**15x**	0.969548	0.962226	34847	0.947313	0.93862	58222
**30x**	0.990916	0.990159	9720	0.980427	0.97953	20567
**60x**	0.996676	0.996115	3702	0.99187	0.991171	8713

b) SNV errors

	HELLO			HELLO (NO HP)		
	**Precision**	**Recall**	**Errors**	**Precision**	**Recall**	**Errors**
**15x**	0.998536	0.995063	21286	0.998263	0.994841	22933
**30x**	0.99947	0.998794	5776	0.999081	0.998744	7239
**60x**	0.999636	0.998794	5223	0.999442	0.998789	5885

Table 2. HELLO vs HELLO without haplotagged data, comparisons for HG001 WGS, PacBio

a) Indel errors

	HELLO			HELLO (NO HP)		
	**Precision**	**Recall**	**Errors**	**Precision**	**Recall**	**Errors**
**15x**	0.964688	0.95011	42985	0.942233	0.925355	66752
**30x**	0.987364	0.986196	13466	0.97639	0.974661	24926

b) SNV errors

	HELLO			HELLO (NO HP)		
	**Precision**	**Recall**	**Errors**	**Precision**	**Recall**	**Errors**
**15x**	0.995558	0.994285	30905	0.995129	0.993913	33340
**30x**	0.996894	0.999524	10934	0.996702	0.999506	11576

DNN architecture

Figure S 3 shows the architecture of HELLO's DNN used for single platform variant calling. CNN_1, CNN_2, and CNN_3 are marked in the figure. Two copies of CNN_1 and CNN_2 are used in the hybrid architecture as explained in the main document, and the features coming from them are combined using the convolutional architecture shown in Figure S 1. The architecture of CNN_3 is the same for single platform and hybrid platform cases. Figure S 2 shows the residual block architecture used in HELLO, which uses two convolutional blocks in series which are short circuited by skip/residual connections. In the case where dimensionality is changed from input to output, the skip connections involve a convolutional layer which matches the output dimensions.

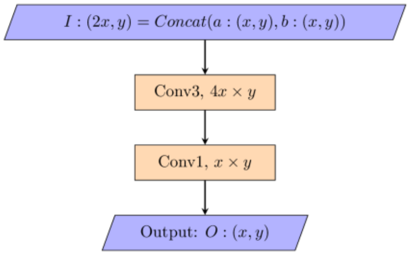

Figure S 1. Module used to fuse features from two different sequencing platforms. The input features are a, b of dimensionality (x, y) each, where x is the number of channels, and y is the length of the channel feature.

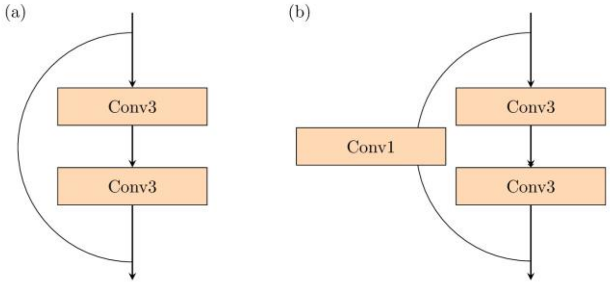

Figure S 2. Residual blocks. (a) Block used when input and outputs have the same dimensions. (b) Block used when input and outputs have different dimensions. The first Conv3 block incorporates the dimensionality changes in this case. Conv1 layer used for dimensionality change doesn't carry activations, but weight normalization is applied.

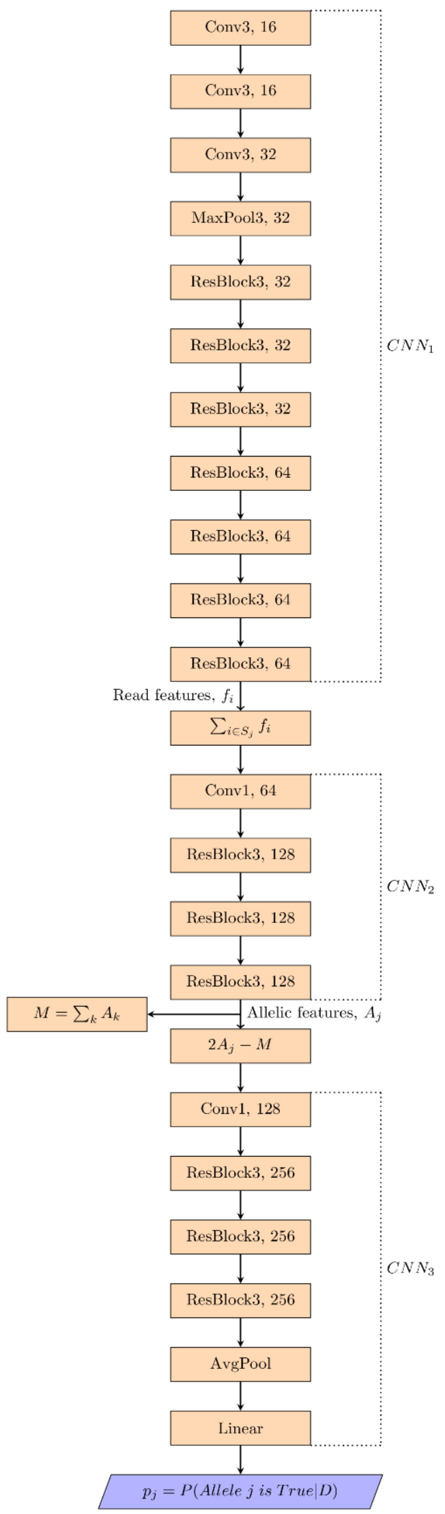

Figure S 3. DNN architecture for single platform variant calling. "<BlockType>P, a" implies a DNN layer/block of type <BlockType> with kernel size P and output channel size a (e.g., Conv3, 16 implies a 1D convolutional layer with 16 channels and kernel size 3). All Conv layers are 1D and have weight normalization applied. Conv layers outside ResBlocks have ReLU activations applied (see previous page regarding ResBlock). Linear layer also has weight normalization applied, and uses sigmoid termination. AvgPool shrinks channel length to 1.

Additional Performance Information

In the main document we compared the DNN run times for HELLO and DeepVariant using a non-standard, three-step HELLO process. Here we provide details on the first and third steps of that flow and compare it to DeepVariant.

- Making DNN inputs (average of 3 runs)
    - DeepVariant: 173s
    - HELLO: 1149.33s
- Post-processing DNN results to produce output VCF file (average of 3 runs)
    - DeepVariant: 54s
    - HELLO: 57s

This indicates that HELLO's data preparation step is the bottleneck. In the data preparation stage, first the alignment file is read to prepare hotspots. Then a second script goes through the hotspots and prepares input data for the DNN. There is duplication of work between these two scripts (e.g., multiple passes through the data) which can be avoided by merging them. In addition, work partitioning between parallel scripts that prepare DNN inputs follows a simple scheme as of now, which is not expected to be load-balanced. This may be rebalanced by improving work partitioning so that the threads do more-or-less equal amount of work. These are two main optimizations that we are considering targeting in a future iteration. We also plan to locate other potential optimizations through profiling of the code.
